# Complexity-Based Measures Inform Effects of Tai Chi Training on Standing Postural Control: Cross-Sectional and Randomized Trial Studies

**DOI:** 10.1371/journal.pone.0114731

**Published:** 2014-12-10

**Authors:** Peter M. Wayne, Brian J. Gow, Madalena D. Costa, C.-K. Peng, Lewis A. Lipsitz, Jeffrey M. Hausdorff, Roger B. Davis, Jacquelyn N. Walsh, Matthew Lough, Vera Novak, Gloria Y. Yeh, Andrew C. Ahn, Eric A. Macklin, Brad Manor

**Affiliations:** 1 Osher Center for Integrative Medicine, Brigham and Women’s Hospital, Harvard Medical School, Boston, Massachusetts, United States of America; 2 Division of Interdisciplinary Medicine and Biotechnology and Margret and H.A. Rey Institute for Nonlinear Dynamics in Medicine, Beth Israel Deaconess Medical Center, Harvard Medical School, Boston, Massachusetts, United States of America; 3 Center for Dynamical Biomarkers and Translational Medicine, National Central University, Chungli, Taiwan; 4 Division of Gerontology, Beth Israel Deaconess Medical Center, Harvard Medical School, Boston, Massachusetts, United States of America; 5 Institute for Aging Research, Hebrew SeniorLife, Roslindale, Massachusetts, United States of America; 6 Movement Disorders Unit, Department of Neurology, Tel Aviv Medical Center, Tel Aviv, Israel; 7 Department of Physical Therapy and Sagol School of Neuroscience, Tel Aviv University, Tel Aviv, Israel; 8 Division of General Medicine and Primary Care, Beth Israel Deaconess Medical Center, Harvard Medical School, Boston, Massachusetts, United States of America; 9 Department of Neurology, Beth Israel Deaconess Medical Center, Harvard Medical School, Boston, Massachusetts, United States of America; 10 Martinos Center for Biomedical Imaging, Massachusetts General Hospital, Boston, Massachusetts, United States of America; 11 Biostatistics Center, Massachusetts General Hospital, Harvard Medical School, Boston, Massachusetts, United States of America; Bielefeld Evangelical Hospital, Germany

## Abstract

**Background:**

Diminished control of standing balance, traditionally indicated by greater postural sway magnitude and speed, is associated with falls in older adults. Tai Chi (TC) is a multisystem intervention that reduces fall risk, yet its impact on sway measures vary considerably. We hypothesized that TC improves the integrated function of multiple control systems influencing balance, quantifiable by the multi-scale “complexity” of postural sway fluctuations.

**Objectives:**

To evaluate both traditional and complexity-based measures of sway to characterize the short- and potential long-term effects of TC training on postural control and the relationships between sway measures and physical function in healthy older adults.

**Methods:**

A cross-sectional comparison of standing postural sway in healthy TC-naïve and TC-expert (24.5±12 yrs experience) adults. TC-naïve participants then completed a 6-month, two-arm, wait-list randomized clinical trial of TC training. Postural sway was assessed before and after the training during standing on a force-plate with eyes-open (EO) and eyes-closed (EC). Anterior-posterior (AP) and medio-lateral (ML) sway speed, magnitude, and complexity (quantified by multiscale entropy) were calculated. Single-legged standing time and Timed-Up–and-Go tests characterized physical function.

**Results:**

At baseline, compared to TC-naïve adults (n = 60, age 64.5±7.5 yrs), TC-experts (n = 27, age 62.8±7.5 yrs) exhibited greater complexity of sway in the AP EC (P = 0.023), ML EO (P<0.001), and ML EC (P<0.001) conditions. Traditional measures of sway speed and magnitude were not significantly lower among TC-experts. Intention-to-treat analyses indicated no significant effects of short-term TC training; however, increases in AP EC and ML EC complexity amongst those randomized to TC were positively correlated with practice hours (P = 0.044, P = 0.018). Long- and short-term TC training were positively associated with physical function.

**Conclusion:**

Multiscale entropy offers a complementary approach to traditional COP measures for characterizing sway during quiet standing, and may be more sensitive to the effects of TC in healthy adults.

**Trial Registration:**

ClinicalTrials.gov NCT01340365

## Introduction

Postural control is critical to the maintenance of balance and avoidance of falls. Balance control systems integrate inputs from the motor cortex, cerebellum, and basal ganglia, as well as feedback from visual, vestibular, and proprioceptive systems to maintain upright posture. When properly functioning, this multi-level neural control system produces stable balance and gait [1], [2]. However, maintenance of balance, even under constant environmental conditions, involves continuous postural adjustments that appear as irregular fluctuations in the center of pressure (COP) displacements recorded from a balance platform [3]–[6]. In the past, these fluctuations were considered to be ‘noise’ and not integral to COP metrics. Thus, quantitative studies of balance focused on average sway or COP parameters, ignoring temporal information. Recent research has demonstrated that COP fluctuations convey important information. In fact, COP dynamics are highly “complex;” i.e., they contain non-random fluctuations that exist across multiple temporal and spatial scales [7], [8]. Complexity-based measures of COP dynamics, including entropy or fractal-based metrics, may therefore be informative outcomes for characterizing age-related decline and frailty [9], fall risk [10] and neuromuscular disorders [1], [6], [11]. This dynamical systems perspective of postural control is also aligned with a growing interest in evaluating multimodal interventions to reduce fall risk, and for complexity-based metrics to assess intervention-related changes in balance system dynamics.

Tai Chi is a multi-component mind–body exercise that is grounded in the holistic model of traditional Chinese medicine. The explicit goals of targeting multiple physiological, motor, and cognitive processes and integrating their dynamics makes Tai Chi particularly well-suited as a multimodal intervention to enhance balance within a systems biology framework [12]. Tai Chi integrates balance, flexibility, and neuromuscular coordination training with a number of cognitive components including relaxation, focused body awareness, imagery, and multi-tasking, that together may result in benefits above and beyond conventional, single modality exercise [13]–[15]. A recent Cochrane review of 159 randomized trials evaluating both exercise and non-exercise interventions reported that Tai Chi trials had a pooled relative risk ratio of 0.71 (95% CI 0.57 to 0.87) for falls [16]. These results are supported by other systematic reviews which conclude that Tai Chi directly reduces fall risk [17], [18] and/or positively impacts factors associated with falling including the fear of falling [19], [20], clinical measures of balance [21]–[23], musculoskeletal strength [22], [23] and flexibility [22], [24].

The effect of Tai Chi on postural control in older adults, however, is less clear. Whereas some studies report reductions in the average speed or magnitude of COP fluctuations beneath the feet when standing [25], [26], others report no change [27]–[30] or even increases [20], [31] in these parameters after Tai Chi training. This is surprising, as postural instability, as evident by excessive or uncontrolled sway, has been identified as one of the key risk factors that lead to falls in many balance-impaired populations [32]–[36]. However, an increase in bodily sway could represent a more dynamic, resilient, and adaptive postural control system; for example in Parkinson’s disease an increase in sway may represent an increase in the range of stability and improvement in postural control [37]. Therefore, better measures of postural control are needed that can quantify the non-linear, dynamic characteristics of postural stability [38].

We have proposed that the impact of Tai Chi on postural control may be better characterized by quantifying its effects on the degree of complexity associated with system output (i.e., COP dynamics) than by traditional sway parameters [12], [14], [39] MSE (multiscale entropy) is one measure of physiologic complexity that is largely independent of traditional metrics and appears to be sensitive to aging and disease [40]. This study performs exploratory analysis of the impact of long- and short-term Tai Chi training on postural control characterized by both MSE and traditional COP measures in a sample of non-randomized Tai Chi expert and randomized Tai Chi naïve healthy older adults. Based on our earlier studies, we predicted that: 1) Tai Chi experts would exhibit greater COP complexity compared to age and gender matched Tai Chi-naïve older adults; 2) Tai Chi-naïve older adults randomly assigned to 6 months of Tai Chi training would exhibit increased COP complexity after training, as compared to a usual care control; and 3) Complexity-based COP measures would be more sensitive to long- and short-term exposure to Tai Chi than traditional sway measures.

## Methods

### Study Design

We employed a hybrid study design that included a two-arm randomized clinical trial (RCT) along with an additional observational comparison group. The RCT evaluated the short-term (6 months) effects of Tai Chi training on healthy community-dwelling older adults. A group of age-matched Tai Chi experts was compared to the randomized Tai Chi and control groups at baseline to evaluate the potential longer-term effects of Tai Chi, and to compare the impact of short- vs. long-term training on balance and functional outcomes. The protocol for this trial and supporting CONSORT checklist are available as supporting information; see S1 Checklist and S1 Protocol. The Institutional Review Board at Beth Israel Deaconess Medical Center (BIDMC) approved this study. The study protocol was first approved in October of 2010. The randomized trial component of this study was registered at ClinicalTrials.gov (NCT01340365). Registration was initiated prior to the start of recruitment, but administrative reassignment of the study staff resulted in a 7-week delay wherein 4 participants were randomized.

### Randomized trial design

**Figure 1:**
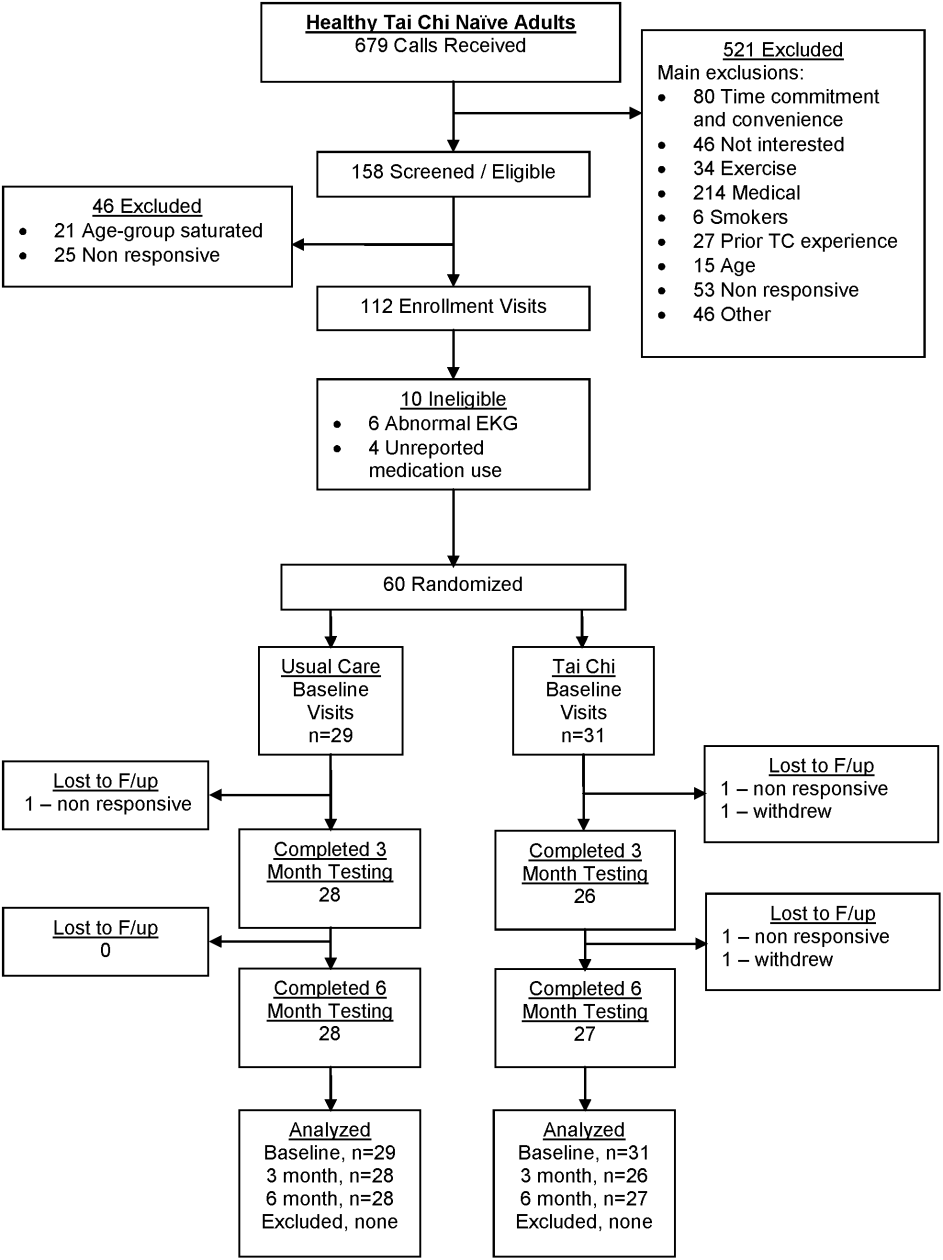
Flow of participants in randomization trial of healthy Tai Chi naïve adults.

A total of 60 healthy older adults, aged 50–79, were randomized to receive 6 months of Tai Chi training in addition to usual health care, or to usual health care alone (control group). Study participants randomized to usual care were offered a 3-month course of Tai Chi as a courtesy following the trial. Randomization was stratified by age (50–59 y, 60–69 y, 70–79 y) and utilized a permuted-blocks randomization scheme with randomly-varying block sizes. Randomization was performed by the study statistician. All outcomes were assessed at baseline, 3 and 6 months; primary staff overseeing assessment and analysis of balance-related and functional assessment outcomes were blinded to treatment assignment. Participant flow through the randomized trial component of the study is summarized in Fig. 1. Recruitment spanned from March 2011 to March 2013. All follow up procedures were completed by September 2013. Further details related to the design of the RCT component of this study are reported elsewhere [12].

### Recruitment for the randomized trial targeting community dwelling healthy adults

Inclusion criteria were: 1) Age 50–79 years; 2) living or working within the Greater Boston area; and 3) willing to adhere to 6 month Tai Chi training protocol. Exclusion criteria were: 1) Chronic medical condition including cardiovascular disease (myocardial infarction, angina, atrial fibrillation or presence of a pacemaker), stroke, respiratory disease requiring daily use of an inhaler; diabetes mellitus; active cancer (diagnosis <5 years ago and requiring ongoing chemotherapy or use of cytotoxic agents), stage III prostate cancer, dermatological cancer with reoccurrence; neurological conditions (e.g., seizure disorder, Parkinson’s, peripheral neuropathy); or significant neuromuscular or musculoskeletal conditions requiring chronic use of pain medication; 2) acute medical condition requiring hospitalization within the past 6 months; 3) self-reported (current) smoking or alcohol/drug abuse; 4) uncontrolled hypertension (resting SBP>160 or DBP>100 mm Hg); 5) abnormal heart rate (resting HR>100 bpm; <50 bpm); 6) abnormal ECG (e.g., supraventricular tachyarrhythmia, atrial fibrillation, significant ST wave abnormality, 2nd and 3rd degree heart block); 6) pregnancy; 7) current use of cardio- or vaso-active drugs and medications that can affect autonomic function including beta agonists and antagonists, drugs with anticholinergic properties (e.g. tricyclic antidepressants or anti psychotics), and cholinesterase inhibitors; 8) self-reported inability to walk continuously for 15 minutes unassisted; 9) regular Tai Chi practice within past 5 years; and 10) regular participation in physical exercise on average 4 or more times per week. Interested individuals underwent both an initial phone screen and an in-person screen at the BIDMC Clinical Research Center. Eligible individuals provided written informed consent and underwent baseline testing prior to randomization.

Participants within both groups were encouraged to follow usual health care as prescribed by their primary physicians, with no limitations set by the study other than those implicit in the eligibility criteria listed above. Participants in the Tai Chi group received 6 months of Tai Chi training in addition to usual care. All Tai Chi interventions were administered pragmatically at one of five pre-screened Tai Chi schools within the Greater Boston area that met specific guidelines described elsewhere [12], [41]. Instructors were asked to teach using the same Tai Chi style, approach and protocols employed for non-study, community participants. Study participants were asked to attend, on average, two classes per week over the 6 month intervention. They were also asked to practice a minimum of 30 minutes, two additional days per week. All schools provided DVDs or printed materials to facilitate home practice. In total, participants were asked to engage in 72 hours of training over the 6 month intervention. Attendance at Tai Chi classes was recorded by instructors and home practice was tracked using a weekly practice log. Participants that reported attending a minimum of 70% of all classes and completing 70% or more of prescribed home practice between each study visit were considered compliant or ‘per protocol’. Adverse events were monitored via forms provided in the participant packet requesting participants to detail the event as well as notifying the study team within 24 hours. Additionally, all Tai Chi schools were given adverse event forms for reporting pre-specified events that occurred during class or at their school. Participants were called monthly by a member of the study team to monitor adverse events as well as to encourage class attendance and home practice.

### Non-randomized comparison groups

#### Tai Chi experts

Twenty-seven healthy older adults (age 50–79 yrs) currently engaged in an active Tai Chi training regimen, and each with over 5 years of Tai Chi practice were recruited for a single observational visit. No limitation was set on Tai Chi style. The Tai Chi expert group was used to evaluate the longer-term effects of Tai Chi training on balance outcomes. Eligibility and screening procedures for Tai Chi experts were identical to those for healthy adults enrolled in the RCT, with the exceptions of no limitation on exercise activity, prior Tai Chi experience, or the use of beta blockers to control diagnosed hypertension.

#### Young Comparison Group

Fifteen healthy, young Tai Chi-naïve adults (age 25–35 yrs) were also recruited for a single observational visit. The group was used to better characterize age-related trends in balance which were required to determine parameters of complexity-based measures of sway as described below.

### Outcome measures

The pre-specified balance-related primary outcome measure for the randomized study was 6-month change in center-of-pressure complexity. All outcome measures were assessed at the Clinical Research Center Syncope and Falls in the Elderly (SAFE) laboratory at Beth Israel Deaconess Medical Center (Boston, MA). Balance related outcomes reported here were part of a larger battery of tests that lasted an average of 3 h (including cardiovascular and gait outcomes to be reported elsewhere).

### Assessment of postural sway

Tests of quiet standing were conducted while subjects stood on a force platform for 60 s with arms by their side, feet shoulder-width apart. Subjects were asked to stand as still as possible and to visually fixate on an “X” drawn on a wall approximately 3 m away at eye-level. COP displacement was recorded with a standard force plate (Kistler Instruments Corp, Amherst, NY). Separate tests were conducted with eyes open and eyes closed. To account for potential trial-to-trial variability, two trials were completed for each condition (random sequence) with at least one minute rest between trials. During the first trial, the position of the hallux was marked with tape to ensure consistent foot placement throughout the study. Both traditional and complexity-based sway parameters were estimated from COP displacement data.

### Traditional COP parameter calculations

Traditional sway parameters included anterior-posterior (AP), medio-lateral (ML), and COP (composite AP and ML) sway velocity (mm/sec), as well as elliptical area (mm^2^). Raw trajectories were extracted from the force plates and smoothed with a 4^th^ order Butterworth low-pass filter with cut-off frequency of 10 Hz [42]. Analyses were performed using Matlab (v7, The Mathworks, Inc., Natick, MA).

### COP complexity calculations

**Figure 2:**
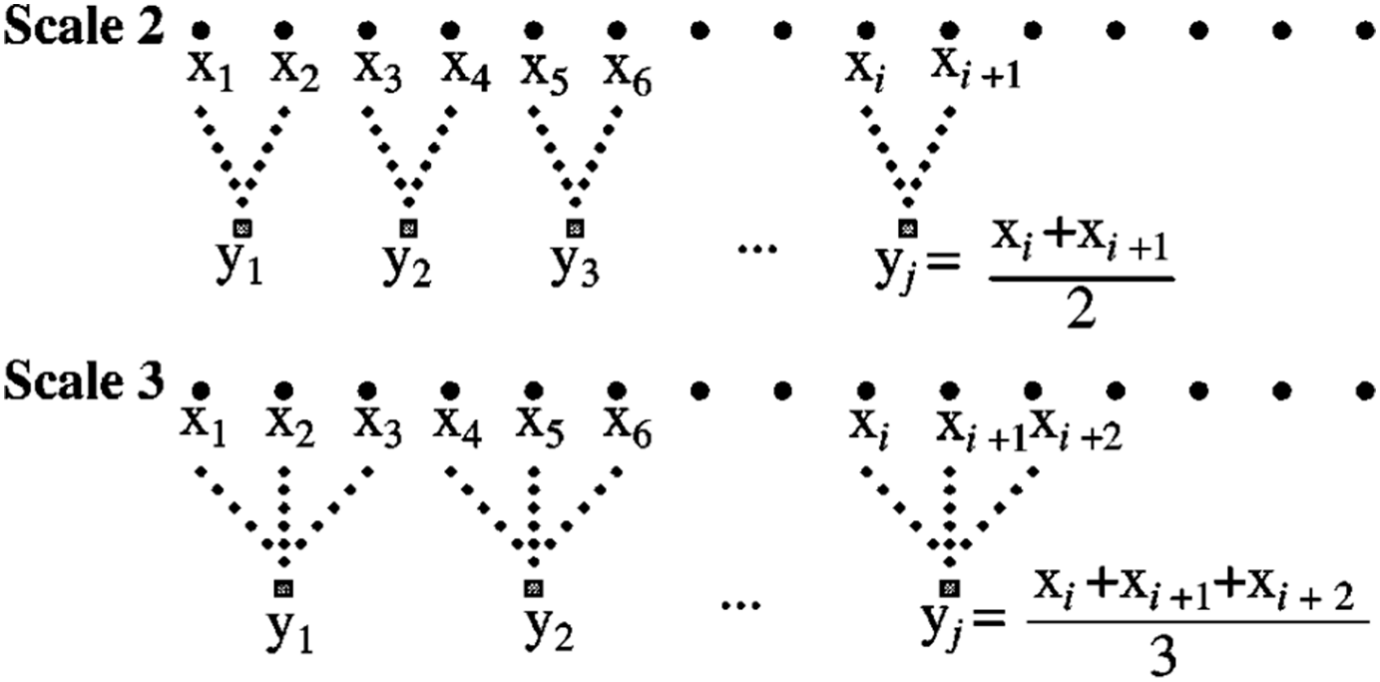
Schematic illustration of the course-graining procedure for multiscale entropy. x_i_ represents the original time-series samples. y_j_ represents the coarse-grained samples at the indicated scale. Adapted from Costa et al. 2002 [72].

COP complexity was calculated using DataDemon (Dynadx Inc., Mountain View, CA) and Matlab. For a given COP time-series, a complexity index, C_I_, was calculated from multiscale entropy (MSE) analysis. Briefly, MSE utilizes Sample Entropy (SampEn) to quantify the degree of irregularity within a time-series across multiple time scales [10]. To do so, a coarse-graining procedure is used, which averages sequential data points to produce multiple new time-series, each with a characteristic time scale. At time scale 1, no coarse-graining occurs and the original N samples are used to calculate SampEn. This metric characterizes irregularity by calculating the negative natural logarithm of the conditional probability that sequences similar for *m* points will remain similar if one more point is added to the sequence. Similar points have amplitudes which fall within a percentage, *r*, of the time-series standard deviation of each other. In our study we used *m* = 2 and *r* = 0.15. At each subsequent time scale factor (τ) in the MSE algorithm, averages of τ points are taken within non-overlapping windows to create a new time-series of length N/τ (Fig. 2). At each time scale, τ, SampEn is calculated to produce an MSE curve (i.e., SampEn versus τ). Using this output, C_I_ was derived by calculating the area under the curve. Since the area under the curve is based on SampEn, a larger area or C_I_ represents greater irregularity over a range of time scales and thus higher complexity. As explained below, the range of scales used to calculate C_I_ varied based on the direction of sway (AP vs. ML) and comparison (long-term or short-term Tai-Chi training). Therefore, we present all complexity results as the complexity index divided by the range of MSE scales for a particular sway direction and comparison. This represents the average C_I_ per MSE scale. We denote this as C_I_/R(τ), where R(τ) represents the range of MSE scales. Normalizing the reported complexity allows comparison across both sway directions and conditions.

### Complexity pre-processing

Raw COP time-series were down-sampled to 250 Hz. Because trends at time scales longer than those being analyzed alter estimates of C_I_, we detrended the COP time-series prior to MSE analysis [10]. To do so, each time-series was detrended using ensemble empirical mode decomposition (EEMD). EEMD is well-suited for the decomposition of nonstationary and nonlinear signals [43]. This process creates multiple intrinsic mode functions (IMFs), each with predominant power over a limited frequency range. While subsequent IMFs may have spectrum overlap, each IMF captures properties of the original time-series at different time-scales [10]. For this analysis, thirteen IMFs were generated. We removed IMFs 9–13 which exhibited frequencies below 0.2 Hz in order to ensure a minimum number of dynamic patterns within the length of our time series [6]. Additionally, we removed IMF 1 and 2 which exhibited frequencies above 20 Hz and are thus unlikely to reflect balance-related biological processes.

### Complexity mode selection

Twenty-one unique IMF combinations were generated, consisting of all continuously sequenced combinations of IMFs 3–8. We then selected the specific IMF combination to be used for analyses based on a statistical comparison between groups prior to treatment, using an approach similar to Wei et al. [44]. Briefly, Wei and colleagues performed statistical tests to choose IMFs that best discriminated young and elderly participants with known differences in balance, and then used these IMF combinations to test the effect of stochastic resonance on the complexity of balance in the elderly group. Similarly, to test our hypothesis that Tai Chi experts would exhibit higher complexity than age-matched controls, we made the a priori assumption that young adults would have higher COP complexity than older adults, and that Tai Chi would restore age-related COP decline. We therefore identified the IMF combination for this hypothesis by comparing the young group and randomized older adult Tai Chi naïve group at baseline. The IMF combination that best distinguished, statistically, the young group from the randomized older adults group was chosen. Table 1 provides a summary of the statistical comparison between these groups at baseline for the IMFs which best distinguished them in each sway direction (AP and ML). We speculate that choosing the IMFs in this manner may identify the time scales of a healthy postural control system since literature shows that young adults generally show superior postural steadiness relative to elderly [45]. These IMFs were subsequently selected for the long-term Tai Chi comparison between Tai Chi experts and age-matched controls of COP complexity. To test the hypothesis that Tai Chi naïve subjects who participated in 6 months of Tai Chi training would improve their complexity relative to age-matched controls, we made the a priori assumption that short-term Tai Chi training would have qualitatively similar effects as long-term training. We thus compared the Tai Chi experts and randomized subjects at baseline, and used the IMF combinations that statistically best distinguished the Tai Chi experts from the randomized group. Table 2 provides a summary of the statistical comparison between these groups at baseline for the IMFs which best distinguished them. In other words, we contend that selecting the IMF combination leading to COP complexity values that best distinguish these two age-matched groups (Experts Vs. Naïve) may be most sensitive to a change in the complexity of postural control within the Naïve group following exposure to short-term Tai-Chi. These IMF combinations were used to compare COP complexity of randomized subjects assigned to Tai Chi against the controls assigned to usual care across their three visits. In both long- and short-term comparisons, we used a two-sample t-test to produce the p-value statistic used for this analysis. All of the comparisons used for IMF selection were done independently for AP and ML directions under the eyes-open condition. The statistically selected time-series of a particular IMF combination was then analyzed using MSE as described above.

**Table 1:** Details of the intrinsic mode functions (IMFs) and MSE scales used for the long-term Tai-Chi comparison between Naïve and Expert groups.

COP Direction	IMFs	Characteristic IMF Frequencies (Hz)	MSE Scales	MSE Scale Range	Young Complexity^a^	Naive Complexity^a^	p-value
AP	3+4+5+6	17.6, 7.7, 3.2, 1.3	1–25	25	1.206±0.256	1.034±0.237	<0.001
ML	3+4+5+6+7	17.9, 8.0, 3.4, 1.2, 0.5	1–35	35	0.943±0.161	0.819±0.181	<0.001

The last three columns show the statistical comparison between the Young and Naive at baseline which led to the selection of these IMFs.

a values provided are mean ± standard deviation with units of complexity index divided by MSE scale range, C_I_/R(τ).

**Table 2:** Details of the intrinsic mode functions (IMFs) and MSE scales used for the short-term Tai-Chi comparison between the randomized to Tai-Chi and Usual Care groups.

COP Direction	IMFs	Characteristic IMF Frequencies (Hz)	MSE Scales	MSE Scale Range	Experts Complexity^a^	Naive Complexity^a^	p-value
AP	5+6+7	3.2, 1.3, 0.6	2–31	30	0.948±0.136	0.843±0.162	<0.001
ML	4+5+6+7+8	8.0, 3.4, 1.2, 0.5, 0.3	1–39	39	0.929±0.207	0.747±0.180	<0.001

The last three columns show the statistical comparison between the Experts and Naive at baseline which led to the selection of these IMFs.

a values provided are mean ± standard deviation with units of complexity index divided by MSE scale range, C_I_/R(τ).

### Clinical measures of balance, physical function and activity

Clinical balance and physical function outcomes were assessed to help interpret sway measures. Single-legged standing balance was assessed according to Vereeck et al. [46]. Three trials were completed under an eyes-closed condition, and the greatest duration (sec) for each condition was used for analysis. This test has been correlated with fall risk in older adults [47]. The Timed Up and Go Test (TUG) was used to quantify functional mobility [48]. TUG has high test-retest reliability and discriminant validity in older adults [49], [50]. Physical activity level was assessed using Physical Activity Status Scale (PASS) [51]. Subjects were asked to estimate their general physical activity during the previous week using an 11-point scale (i.e., 0–10). The scale quantifies physical activity duration by a combination of the minutes of exercise per week and the intensity of this exercise (heavy, modest, or none). The concurrent validity of the scale has been documented in both men and women and scores correlate with maximal oxygen consumption in younger and older adults [52], [53].

### Statistical methods

Baseline measures of Tai Chi masters and Tai Chi naïves were compared in a linear model controlling for age. The group difference and adjusted means for a participant with mean age were estimated. Trends in balance measures over the 24-week intervention were compared between naïve participants randomized to Tai Chi or usual care using a random-slopes model with shared baseline. All longitudinal analysis were conducted according to the intention-to-treat paradigm. The model included fixed effect of time, time×treatment, age, and time×age and random participant-specific intercepts and slopes with unstructured covariance. The shared baseline assumption, enforced by omitting a treatment main-effect term, properly reflects the true state of the population sampled prior to randomization and has the advantage of adjusting for any chance differences at baseline in a manner similar to ANCOVA. A similar model with visit treated as a categorical term was also fit to explore possible non-linearity in the time profiles. As above, treatment-group differences and adjusted means for a participant with mean age were estimated as well as their 95% confidence intervals. Residual diagnostics were used to test assumptions of data normality, and in a small number of cases where non-normality was detected, log-transformation was employed. Log-transformation had only minor impacts on statistical results and yielded equivalent inference, thus only non-transformed results are presented. Associations between percent changes in traditional and complexity-based measures of balance vs. dosage of Tai Chi training, and functional measures vs. Tai Chi training dosage were estimated by linear regression. All inferential tests were two-tailed at alpha = 0.05. We chose to report comparison-wise p-values without adjustment for multiple comparisons to avoid inflating type II errors, recognizing that the nominal p-values underestimate the overall experiment-wise type I error rate. Our results are intended as hypothesis generating, not definitive tests of efficacy for Tai Chi on any specific measure of balance. All analyses were performed in SAS (version 9.3, SAS Institute, Cary, NC).

Regarding sample size considerations, for cross-sectional comparisons, we estimated that a sample size of 27 Tai Chi Expert and 60 Tai Chi naïve subjects would provide power to detect an effect size of 0.63. For the randomized trial, we estimated that the sample of 60 participants randomized 1∶1, the study would have 80% power to detect a main effect of treatment if the true effect size was at least 0.74 based on a two-tailed test at p<0.05.

## Results

### Participant characteristics

Tai Chi experts (n = 27) reported an average of 24.6±12 years of Tai Chi training experience (median 20 yrs, range 10–50 yrs). Approximately equal numbers reported Yang (n = 12) and Wu (n = 15) style Tai Chi as their primary training systems; however, all reported having training experience in others styles of Tai Chi, related internal and external martial arts (e.g. kung fu, bagua) and/or mind-body practices (e.g. yoga, meditation). Sociodemographic characteristics of the Tai Chi experts were well-matched with the older Tai Chi naïve group with respect to average age and global cognitive status. Compared with naïve older controls, experts included a slightly greater proportion of men and Asians, and had lower BMI’s and higher levels of physical activity (Table 3).

**Table 3:** Participant characteristics for observational and randomized groups.

	Observational Groups			Randomized Groups and Sub-groups		
	Older Tai Chi Experts	Older Tai Chi Naïve	Young Tai Chi Naïve	Usual Care	Tai Chi	TC Compliant
	(n = 27)	(n = 60)	(n = 15)	(n = 29)	(n = 31)	(n = 15)
**Age** a	62.78±7.57	64.18±7.68	28.73±3.20	64.45±7.42	63.94±8.02	63.47±6.98
**Gender** n(%)						
Male	13 (48.1%)	20 (33.3%)	8 (53.3%)	11 (37.9%)	9 (29%)	5 (33.3%)
Female	14 (51.9%)	40 (66.7%)	7 (46.7%)	18 (62.1%)	22 (71%)	10 (66.7%)
**Race** n(%)						
White	22 (81.5%)	55 (91.7%)	15 (100%)	26 (89.7%)	29 (93.5%)	15 (100%)
African American	1 (3.7%)	3 (5%)	0 (0%)	3 (10.3%)	0 (0%)	0 (0%)
Asian	4 (14.8%)	2 (3.3%)	0 (0%)	0 (0%)	2 (6.5%)	0 (0%)
**Ethnicity** n(%)						
Non-Hispanic/Non-Latino	26 (96.3%)	59 (98.3%)	14 (93.3%)	29 (100%)	30 (96.8%)	14 (93.3%)
Hispanic/Latino	1 (3.7%)	1 (1.7%)	1 (6.7%)	0 (0%)	1 (3.2%)	1 (6.7%)
**Education (yrs)** a	18.44±3.34	16.7±3.25	17.93±2.52	16.19±3.03	17.13±3.41	17.43±2.85
**MMSE (out of 30)** a	29.07±1.11	29.12±1.01	n/a	29.21±0.82	29.03±1.17	28.93±1.28
**BMI (kg/m^2^)** a	23.54±2.35	26.46±5.46	25.43±3.21	26.54±5.83	26.38±5.19	26.81±4.60
**Physical Activity Level** a **^,^** b	6.0±2.0	4.4±2.2	n/a	4.0±2.0	4.0±2.0	4.9±2.2
**Single leg stance time (eyes closed) (sec)** a	19.6±14.07	8.56±6.27	24.82±15.23	7.52±5.74	9.53±6.67	8.57±5.79
**Timed Up and Go (sec)** a	5.07±0.86	5.94±1.14	4.43±0.68	5.68±0.99	6.17±1.24	6.11±1.11

a values provided are mean ± standard deviation.

b Physical Activity Level descriptions:

4 = Run about 1 mile per week OR walk about 1.3 miles per week OR spend about 30 minutes per week in comparable physical activity.

5 = Run about 1 to 5 miles per week OR walk 1.3 to 6 miles per week OR spend 30 to 60 minutes per week in comparable physical activity.

6 = Run about 6 to 10 miles per week OR walk 7 to 13 miles per week OR spend 1 to 3 hours per week in comparable physical activity.

Tai Chi naïve adults subsequently randomized to Tai Chi plus usual care vs. usual care alone were comparable at baseline. For all variables, values for the subset of participants that were found to be ‘per-protocol’ were comparable to those in the larger sample thereby minimizing potential sources of bias in post-hoc comparisons between control and Tai Chi compliant groups.

### Recruitment and protocol adherence

Six hundred and seventy-nine older adults were approached in order to recruit and enroll 60 eligible healthy adults. The majority were excluded due to medical ineligibility (214) and limited time availability or interest (179). Of those enrolled, 97% (28/29) and 87% (27/31) of individuals in the usual care and Tai Chi group completed the primary 6-month follow-up assessment, respectively. All 60 participants completing baseline assessments were included in intent to treat analyses. Adherence to the Tai Chi protocol was variable. Fifteen of the 31 (48%) participants in the Tai Chi group were protocol compliant, attending 70% of classes and completing 70% of required home practice between each study visit. This compliant subgroup had a mean exposure to Tai Chi training of 89.3 hours; in comparison the intent to treat group had a mean exposure of 60.9 hours. Overall satisfaction with Tai Chi programs, assessed on a 1–7 scale (1 is best score) were high, with a mean ± standard deviation rating of 1.6±1.1 at 3 months and 1.7±1.4 at 6 months.

A total of 4 non-serious adverse events were reported throughout study. All events were reported by participants randomized to the Tai Chi group. Only 2 of the 4 events were determined to be related to the Tai Chi intervention (both minor musculoskeletal injuries (one wrist, one ankle)).

### The association between long-term Tai Chi training and standing postural control

**Figure 3:**
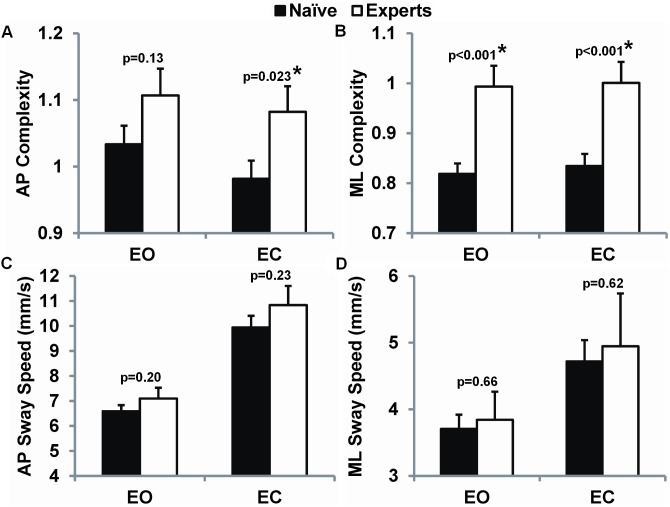
Complexity-based and traditional measures of sway for age-matched Tai Chi naïve and Tai Chi expert older adults. Mean value and standard errors for sway measured in the anterioposterior (AP) and mediolateral (ML) planes and during eyes-open (EO) and eyes-closed (EC) conditions. Statistical comparisons were adjusted for age.

Complexity-based measures of both anterior-posterior (AP) and mediolateral (ML) sway assessed under eyes open (EO) and eyes closed (EC) conditions were consistently higher for Tai Chi experts vs. Tai Chi naïve subjects (Table 4). Age adjusted linear models revealed that average MSE of sway in the Tai Chi experts group were markedly and statistically greater than the Tai Chi naïve group under ML EO (21%; p<0.001) and ML EC (20%; p<0.001) testing conditions, and to a lesser degree in the AP EC (6%; p = 0.023) condition (see Fig. 3 a, b). A negative overall trend with age was only observed in the AP EC condition but there were no group×age interaction effects (Table 4).

**Table 4:** Complexity-based and Traditional measures of sway for Naïve and Expert groups at baseline.

Outcome	Groups		Statistical Result	
	Naïve (n = 60)	Expert (n = 27)		
Complexity COP Measures	Mean ± S.D.^a^	Mean ± S.D.^a^	p - age^b^	p – group^c^
AP - Eyes Open (C_I_/R(τ))	1.033±0.216	1.107±0.209	0.510	0.131
AP - Eyes Closed (C_I_/R(τ))	0.982±0.211	1.082±0.200	**0.028**	**0.023**
ML - Eyes Open (C_I_/R(τ))	0.819±0.161	0.993±0.217	0.353	**<0.001**
ML - Eyes Closed (C_I_/R(τ))	0.834±0.188	1.001±0.219	0.574	**<0.001**
**Traditional COP Measures**				
AP Sway Speed - Eyes Open (mm/s)	6.59±1.90	7.09±2.26	**0.040**	0.203
AP Sway Speed - Eyes Closed (mm/s)	9.94±3.61	10.83±3.99	**0.050**	0.227
ML Sway Speed - Eyes Open (mm/s)	3.71±1.65	3.84±2.19	0.143	0.656
ML Sway Speed - Eyes Closed (mm/s)	4.72±2.46	4.95±4.12	**0.050**	0.624
COP Sway Speed - Eyes Open (mm/s)	8.29±2.45	8.85±3.21	**0.037**	0.276
COP Sway Speed - Eyes Closed (mm/s)	11.97±4.29	12.92±6.31	**0.028**	0.296
Elliptical Area - Eyes Open (mm^2^)	161.8±136.5	170.4±332	0.083	0.748
Elliptical Area - Eyes Closed (mm^2^)	200.3±135.6	166.4±126.8	0.148	0.330

a values provided are mean ± standard deviation.

b p-values for testing for an effect of age.

c p-values for comparing Naïve vs. Expert groups after adjusting for age.

In contrast, we found no differences between the Tai Chi naïve and Tai Chi expert group for traditional measures of sway, although 7 of 8 traditional measures exhibited trends towards greater sway among Tai Chi experts (Table 4). More than half of the traditional parameters increased significantly with age (age P≤0.05) and AP EO sway speed, AP EC sway speed and COP EC sway speed exhibited a significant group×age interaction indicating a larger Tai Chi-related difference among older participants. Adjusting for baseline BMI and exercise activity in linear models did not substantially alter the results of the complexity measures for ML EO or EC, but did modestly reduce significance levels for AP EC complexity (p = 0.147, p = 0.086 for BMI and exercise activity, respectively). Education level, MMSE, and TUG did not impact any model results.

### The effect of short-term Tai Chi training on standing postural control

**Figure 4:**
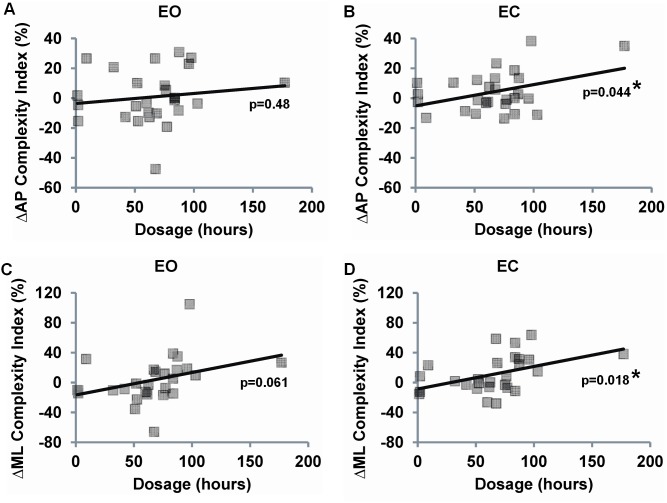
Associations between changes in both complexity-based (A, B) and traditional measures (C, D) of sway vs. Tai Chi dosage (total hours practiced) among Tai Chi naïve individuals exposed to six months of training. Percent changes in sway were assessed between the baseline and 6 month follow up visit for the anterioposterior (AP) and mediolateral (ML) planes and during eyes-open (EO) and eyes-closed (EC) conditions. Analyses excluding the largest x-axis value (i.e. potential outlier) reduced ΔAP EC p-value to a non-significant level (p = 0.423), however, the p-value for the ΔML EC was less affected (p = 0.037).

Intention-to-treat analyses using age adjusted linear models revealed no statistically significant group×time interactions for any complexity-based measures of sway, although complexity assessed under the EC condition trended towards higher values in the Tai Chi group (Table 5). Analyses limited to per-protocol Tai Chi subjects indicated a significantly greater increase in complexity in the ML EC condition in the Tai Chi group compared to usual care (P = 0.029). Additionally, associations between changes in complexity score and Tai Chi dosages (combined total hours of class and home training) were all positive and statistically significant under AP EC and ML EC conditions (Fig. 4a–d). Of note, sway complexity levels following 6 months of Tai Chi training did not reach the complexity levels of Tai Chi experts under all testing conditions (P<0.03 for all measurements).

**Table 5:** Complexity-based and Traditional measures of sway for the Randomized to Tai-Chi and Randomized to usual care groups across visits.

COP Outcome Measures	Groups								Statistical Result	
	Tai-Chi			Usual Care			Tai-Chi	Usual Care		
	Baseline	3 Months	6 Months	Baseline	3 Months	6 Months	Across Visit	Across Visit		
Complexity	Mean ± S.D.^a^	Mean ± S.D.^a^	Mean ± S.D.^a^	Mean ± S.D.^a^	Mean ± S.D.^a^	Mean ± S.D.^a^	Slope (95% CI)^b^	Slope (95% CI)^ b^	p - age^c^	p –grp*t^d^
AP - EO (C_I_/R(τ))	0.831±0.124	0.843±0.123	0.824±0.148	0.857±0.167	0.845±0.171	0.811±0.163	−0.008 (−0.031, 0.015)	−0.017 (−0.039, 0.006)	0.951	0.499
AP - EC (C_I_/R(τ))	0.835±0.156	0.865±0.176	0.857±0.145	0.878±0.129	0.863±0.138	0.846±0.127	0.007 (−0.011, 0.025)	−0.010 (−0.028, 0.008)	0.070	0.117
ML - EO (C_I_/R(τ))	0.706±0.167	0.731±0.202	0.686±0.171	0.792±0.162	0.757±0.161	0.742±0.159	−0.019 (−0.046, 0.007)	−0.012 (−0.038, 0.014	0.580	0.605
ML - EC (C_I_/R(τ))	0.743±0.177	0.806±0.215	0.787±0.175	0.838±0.160	0.826±0.158	0.777±0.138	0.007 (−0.019, 0.033)	−0.013 (−0.038, 0.013)	0.914	0.166
**Traditional**										
AP SS - EO (mm/s)	6.33±1.72	6.86±2.35	6.75±1.98	6.86±2.07	6.76±2.07	6.72±2.16	0.119 (−0.124, 0.361)	−0.006 (−0.247, 0.235)	0.497	0.432
AP SS - EC (mm/s)	10.00±3.97	10.57±3.76	10.77±4.53	9.88±3.25	9.98±3.13	9.88±3.13	0.295 (−0.075, 0.666)	0.033 (−0.334, 0.400)	0.744	0.289
ML SS - EO (mm/s)	3.73±1.54	3.81±1.44	3.96±1.88	3.68±1.79	3.33±1.45	3.55±1.46	0.154 (−0.032, 0.340)	−0.054 (−0.238, 0.129)	0.479	0.077
ML SS - EC (mm/s)	4.92±2.55	4.61±1.72	4.97±2.66	4.50±2.40	4.05±1.76	4.11±1.59	0.105 (−0.147, 0.356)	−0.202 (−0.451, 0.046)	0.317	**0.034**
COP SS - EO (mm/s)	8.10±2.15	8.60±2.85	8.63±2.79	8.49±2.75	8.19±2.60	8.31±2.51	0.223 (−0.084, 0.530)	−0.027 (−0.330, 0.277)	0.432	0.213
COP SS - EC (mm/s)	12.15±4.76	12.44±4.07	12.88±5.40	11.78±3.80	11.56±3.63	11.53±3.35	0.352 (−0.088, 0.791)	−0.095 (−0.530, 0.341)	0.537	0.119
Ellip. Area - EO (mm^2^)	172.7±125.1	217.6±211.1	250.5±270.7	150.2±149.1	137.6±147.4	141.1±103.9	35.9 (5.09, 66.8)	1.88 (−28.7, 32.5)	0.596	0.051
Ellip. Area - EC (mm^2^)	234.3±140.5	213.7±177.8	287.4±275.7	163.9±122.2	154.1±92.6	173.8±100.2	33.3 (3.20,63.4)	1.07 (−29.1, 31.2)	0.226	0.078

a values provided are mean ± standard deviation. ^b^values are estimated slope (lower 95% confidence interval, upper 95% confidence interval). ^c^p-values for testing for an effect of age indicated by p-age. ^d^p-values for comparing mean rates of change among Tai-Chi vs. usual care groups indicated by p-group*time.

Age adjusted linear models indicated no statistically significant group×time interactions for 7 of the 8 traditional sway parameters, but as with cross-sectional comparisons of Tai Chi experts vs. Tai Chi naïves, values for all 8 parameters showed trends towards increasing sway with exposure to Tai Chi (Table 5). These trends were not affected by limiting analyses to compliant per-protocol Tai Chi subjects. Adding physical activity into longitudinal models for both traditional and complexity based COP measures did not significantly affect results. Of note, in contrast to complexity measures, values of most traditional sway parameters following 6 months of Tai Chi were very similar to values of Tai Chi experts.

### Impact of short- and long-term training on physical function

**Figure 5:**
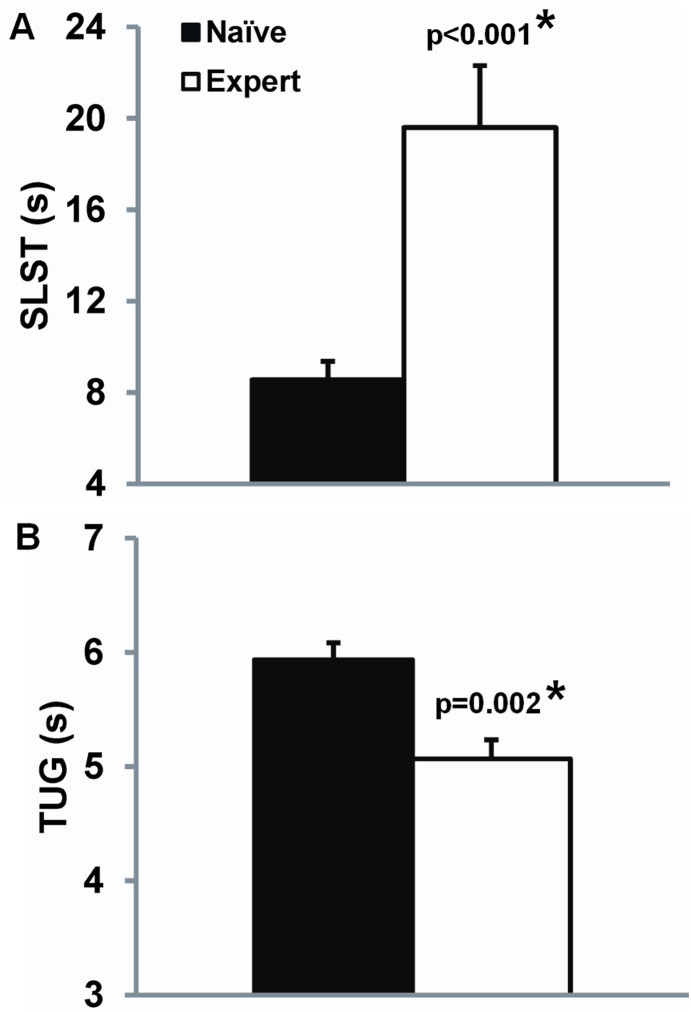
Measures of physical function for age-matched Tai Chi naïve and Tai Chi expert older adults. Mean value and standard errors for A) Single leg stance time (SLST) and B) timed up and go (TUG). Comparisons were adjusted for age.

**Figure 6:**
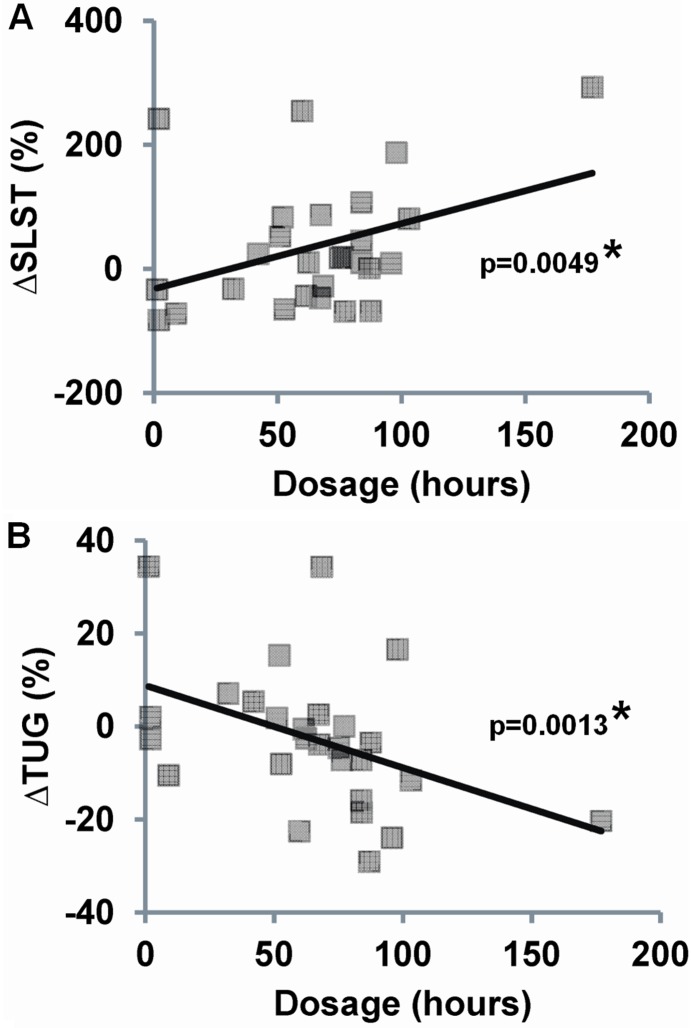
Associations between changes in physical function and Tai Chi dosage (total hours practiced) among Tai Chi naïve individuals exposed to six months of training. Percent changes in function were assessed between the baseline and 6 month follow-up visit for single leg stance time (SLST) and timed up and go (TUG) parameters. Analyses excluding the largest x-axis value (i.e. potential outlier) reduced ΔSLST to a non-significant level (p = 0.286), however, the p-value for the ΔTUG was less affected (p = 0.0062).

Both single leg stance time (SLST) and Timed Up and Go (TUG) performance were better in the Tai Chi expert group as compared to the Tai Chi naïve group (P<0.001; Fig. 5). Changes over 6 months in functional performance showed trends towards being greater in those randomized to Tai Chi vs. usual care, but group×time interactions were not significant in either intent-to-treat or per-protocol analyses. However, associations between changes in functional scores vs. Tai Chi dosage (combined total hours of class and home training) were positive and statistically significant for both SLST and TUG (Fig. 6). Of note, levels of physical function following 6 months of Tai Chi training were lower than that seen in the Tai Chi experts (p = 0.064 and p = 0.001 for SLST and TUG, respectively).

## Discussion

Maintaining balance and preventing falls among older people is an urgent public health challenge worldwide with significant direct and indirect costs to society [54]–[56]. This challenge has led to extensive efforts to improve our understanding of the complex physiology underlying age-related decline of postural control, to identify interventions to reduce the risk of falling in older adults, and to develop markers to accurately measure the short- and long-term impact of interventions targeting balance and fall risk. Mounting evidence suggests that Tai Chi is a promising intervention for reducing falls, especially in older (transitionally) frail and neurologically impaired adults that are at high risk of falling [16], [37], [57], [58]. However, less is known about Tai Chi’s impact on healthy adults that are at a lower risk for falling, the relevance of measures of sway for evaluating intervention-related impacts on postural control, and the mechanisms through which Tai Chi may reduce future fall risks.

To our knowledge, this study represents the first randomized trial evaluating the impact of Tai Chi on complexity-based measures of postural sway. Our main finding is that complexity measures may provide a complementary, alternative and perhaps more discriminating metric for characterizing postural sway during quiet standing and the impact of complex mind-body interventions such as Tai Chi on balance. Among healthy adults, long-term exposure to Tai Chi training was generally associated with increased sway complexity. Intent-to-treat analyses did not indicate a significant effect of shorter-term (6 month) exposure to Tai Chi training on sway complexity or function. However, post-hoc analyses indicated positive and significant associations between hours of exposure to Tai Chi training and increases in sway complexity and functional performance. In combination with prior research linking enhanced complexity with health (discussed below), these findings suggest that MSE may be a sensitive maker for characterizing the benefits of Tai Chi to postural control during quiet standing in healthy adults. In contrast, traditional measures of sway did not reflect obvious effects of either long- or short-term Tai Chi training. In fact, although trends were not statistically significant, the majority of traditional sway outcomes increased with exposure to Tai Chi. Increased sway in many, but not all (see below), populations has been associated with increased fall risk [32]–[36]. Our findings suggest that these traditional sway metrics may be relatively insensitive to the effects of Tai Chi in healthy active older adults, and may also require alternative interpretations.

The findings in the current study parallel those of a pilot observational study evaluating the effects of 24 weeks of Tai Chi for individuals with peripheral neuropathy. Tai Chi training significantly increased MSE-derived complexity of standing COP dynamics, and complexity indices were associated with improved plantar sensation and physical function [39]. In contrast, no intervention-related changes in traditional sway parameters were observed.

Although higher values of traditional sway metrics have been identified as one of the key risk factors that lead to falls [32]–[36], the effects of Tai Chi on traditional sway metrics is variable and unclear. Some studies report reductions in the average speed or magnitude of COP fluctuations [25], [26], [59], others report no change [27]–[30], and similar to the trends we observed in this study, some studies have reported increases [20], [31] in traditional sway parameters after Tai Chi training. Of note, the effects of conventional therapies on COP metrics have been variable, sometimes increasing following interventions [60]. Some of this variability may result from the use of testing conditions that vary from quiet standing to more provocative challenges to balance. It is also possible that this reported variability in the effects of Tai Chi on traditional sway parameters, and perhaps also the insensitivity in response to Tai Chi we observed in the present study, reflects a diversity of strategies for postural control and associated reductions in fall risks. A hallmark study by Wolf and colleagues [61] compared the effect of 15 weeks of computerized balance training to Tai Chi on standing postural sway and fall risk in older frail adults. Balance training, but not Tai Chi, reduced the average magnitude and velocity of sway. Under certain conditions, average sway magnitude was actually greater following Tai Chi training. Interestingly, however, the risk of falling was significantly reduced only in the Tai Chi group (adjusted RR 0.51 vs. 0.98 for Tai Chi and balance training, respectively). Wolf and colleagues [20] suggested that Tai Chi-related changes in fall risk may be due to factors other than average sway characteristics. They also suggested that the inherent aim of Tai Chi training might not be to reduce sway, but rather to provide corrective skills and improve confidence for managing postural instability. Other studies also support that Tai Chi may increase the limits of stability within which individuals are comfortable [31], [37], [62], [63]. Finally, it is possible that trends towards higher sway may be related to reduced musculoskeletal rigidity, as evidence supports that Tai Chi training reduces muscle co-contraction, and increases flexibility [64], [65]. This could produce beneficial effects in conditions such as Parkinson’s disease [37]. This perspective is indirectly supported by studies of individuals with ligament laxity and hypermobility (Ehlers-Danlos syndrome) who exhibit markedly greater COP sway ranges when compared to healthy controls [66]. The same study also reported that complexity based metrics of COP (approximate and sample entropy) were markedly lower in the hypermobile group, interpreted as a lower level of integrated postural control. Together, these results suggest that the non-linear measures we explored here, which focus on quantifying the moment-to-moment control of postural sway during relatively unchallenged quiet standing conditions, may afford unique and complementary insight into Tai Chi’s effect on mechanisms of postural control that are not well-captured by traditional measures.

There is growing evidence to suggest that greater complexity of sway is associated with higher levels of function and health. One recent study [9] evaluated the COP dynamics from 550 participants (avg. 77.9 yrs) whose frailty phenotype (not frail, pre-frail, or frail) was determined using standard criteria. Complexity of COP dynamics was quantified using MSE. Compared to the non-frail, MSE of COP was lower in pre-frail and frail groups (p<0.002). Although traditional linear measures of balance (root mean square amplitude of sway) were also associated with frailty, statistical models indicated that only MSE independently predicted frailty status after accounting for other physiologic determinants of balance such as age, vision, lower extremity strength, peripheral neuropathy, and frontal-executive cognitive function. MSE apparently quantifies an aspect of frailty not captured by other measures.

Complexity-based analyses of COP have been shown to be sensitive indicators of pathology. Cavanaugh et al. [67] used approximate entropy (ApEn) to examine changes in postural stability in 27 collegiate athletes following cerebral concussions (all underwent baseline testing prior to their athletic season and their injury). COP dynamics were also measured in a cohort of age-matched healthy non-athletes. None of the athletes with concussions exhibited any indications of postural instability using standard testing. However, ApEn values, a measure similar to SampEn, following concussions generally declined, whereas ApEn values remained stable for healthy subjects. Complexity markers are now being considered as part of a post-concussion assessment protocol [5].

A number of studies have shown that the complexity of COP time series declines with age [4], [10], [68], [69]. For example, Costa et al. [10] evaluated short-term COP dynamics in 15 healthy young adults (avg. 27 y), 22 healthy elderly adults (avg. 75 y), and 22 elderly fallers (avg. 74 y). For sway velocity measures, complexity indices in both the AP and ML plane were significantly different for all three groups. In the current study, we did not observe any significant age or treatment×age effects on complexity. This may reflect a lack of association between MSE measures of sway and aging, or it may reflect a bias in our study sampling design. Because our eligibility criteria for Tai Chi naïve older adults was quite narrow with respect to acceptable comorbidities and use of medications, our sample reflects a very healthy adult population. As the number of individuals that met our eligibility criteria decreased with age, those in the oldest stratified age group (70–79 yrs) were more likely to represent extremely healthy individuals, whereas those on our younger group (50–59 yrs) that met our eligibility criteria were more likely to be of average health. This interpretation is supported by our TUG data. Average TUG scores for our 60–70 and 70–80 y group were 6.1 and 6.2 seconds; in comparison, normative data for ambulatory adults in these two age groups is 8 and 9 seconds. Due to this bias, our ability to evaluate the direct effects of age on COP complexity, as well as how age may impact response to Tai Chi is partially limited.

### Strengths and Limitations

There are a number of strengths and limitations to this study. One strength is our hybrid design and our ability to compare long- and short-term training of Tai Chi. Multiple prior cross-sectional studies have compared older Tai Chi experts vs. older Tai Chi naïve adults with respect to traditional measures of balance and function [70], [71]. However, to our knowledge, our study is the first to compare the effects of short-term Tai Chi training to long-term training, using both traditional and novel measures of postural control, and employing a prospective randomized trial design with identical protocols to our cross-sectional study. Our hybrid design also has important limitations. Samples for both our cross-sectional comparisons and our RCT were small, and could have resulted in type II errors. Additionally, as with any cross-sectional study, comparisons between Tai Chi experts and Tai Chi naïve may be confounded by differences between groups other than exposure to Tai Chi. While linear models that included potential confounders (e.g., BMI, exercise activity) suggested an independent effect of long-term Tai Chi even after these factors were taken into account, other factors, including training in other mind-body exercises (qigong) and martial arts could not be fully accounted for. For these reasons, caution should be used when attributing any cross-sectional differences solely to the effect of long-term Tai Chi training. The lack of an active exercise control in our RCT also leaves open the possibility that the effects we observed were primarily due to psychosocial interactions. Future studies comparing Tai Chi vs other exercise controls on complexity measures of sway will be informative. Future comparative studies should also consider combining detailed measures of physiological processes known to impact postural control with complexity based measures. This approach would facilitate the understanding of relative contributions of specific physiological processes and their relationship to specific IMFs, and thus the pathways through which interventions like Tai Chi impact balance. Finally, in this exploratory study, we did not make statistical adjustments for multiple comparisons, which increases the likelihood that we observed some positive treatment related effects simply due to chance.

## Conclusions

This study represents the first evaluation of the utility of complexity-based measures of postural sway for characterizing the impact of short- and long-term mind-body exercise training, namely Tai Chi. Multiscale entropy offers a complementary approach to traditional COP measures for characterizing sway during quiet standing, and may be more sensitive to the effects of Tai Chi in healthy adults. Future appropriately powered studies should explore how complexity vs. traditional measures of sway correlate with validated measures of functional performance and help predict critical public health outcomes including long-term risk of injurious falls.

## Supporting Information

S1 Checklist **CONSORT Checklist.**
(DOC)

S1 Protocol **Trial Protocol.**
(DOCX)
