# Supplementary material for: Complexity-Based Measures Inform Effects of Tai Chi Training on Standing Postural Control: Cross-Sectional and Randomized Trial Studies
Source: PLoS One. 2014 Dec 10;9(12):e114731. doi: 10.1371/journal.pone.0114731 (PMC4262457; doi:10.1371/journal.pone.0114731)
Supplement: S1 Protocol — Trial Protocol. (DOCX) [file pone.0114731.s002.docx]

PART B

**STUDY DESCRIPTION**

| **Title of Protocol** | Tai Chi, Physiologic Complexity, and Healthy Aging | | |
| --- | --- | --- | --- |
| Principal Investigator | Gloria Y. Yeh, MD | | |
| Co-Investigators | Peter Wayne PhD, Lewis Lipsitz MD, Vera Novak MD, Brad Manor PhD, Andrew Ahn MD, Roger Davis ScD, Madelena Costa PhD, Jeff Hausdorff PhD, Mary Teresa Quilty MSc, Ary Goldberger MD, Matthew Lough BS, Jacquelyn N. Walsh BS, Danielle L. Berkowitz BS, Matthew L. Holmes BS, Brian Gow BS, Eric A. Macklin PhD | | |
| Mailing Address | 1309 Beacon Street Brookline, MA, 02446 | | |
| E-Mail Address | gyeh@bidmc.harvard.edu | | |
| P.I.’s Telephone | 617-754-1419 | P.I.’s Pager | Fax: 617-754-1446 |
| Sponsor/Funding Source | NIH | | |

# B1. PURPOSE OF PROTOCOL

| To evaluate the effects of Tai Chi—a mind-body exercise––on age-related loss of physiological complexity (using fractal and entropy based measures), and to understand the relationship between complexity, function and adaptability, we will conduct a two-arm prospective randomized clinical trial. Our overarching goal is to evaluate if six months of Tai Chi training, compared to a waitlist control receiving standard medical care, can enhance physiological complexity and adaptability in older Tai Chi-naïve adults. Secondary goals of the study are to characterize the relationship between complexity biomarkers, measures of function, and resilience. This pilot study will inform a future more definitive trial by providing information on recruitment and retention, compliance, dose-dependent effects, preliminary estimates of effect size, and the optimal biomarkers of complexity, function, and adaptive capacity.    **Specific Aim #1: To determine if 6 months of Tai Chi training can increase complexity, function, and adaptive capacity of multiple physiological systems in older healthy adults.** Sixty healthy older adults (50-79y) naïve to Tai Chi will be recruited from the Boston community and randomized to either 6 months of Tai Chi training or to a waitlist control. Measurements of complexity, adaptive capacity, and function will be assessed at baseline, 3, and 6 months. Dynamics of cardiovascular (heart rate), balance (center-of-pressure displacement), and locomotor (gait stride interval) systems will be quantified using indices derived from two complementary analytic methods developed by members of our team––detrended fluctuation analysis^26^ and multi-scale entropy.^6^ Function will be characterized by exercise capacity, clinical measures of balance, lower and upper extremity strength, cognitive function, and health related quality of life. Adaptability of multiple physiological systems will be assessed using common perturbations including: heart rate responses to posture change, center of pressure (i.e. balance) responses to a dual cognitive task and closed eyes, and c) gait responses to dual cognitive task. In addition to informing the feasibility of a future trial, outcomes will provide preliminary data to evaluate the following hypotheses.  ***Hypotheses*** *Compared to the control group:*  *H.1.a) Those who participate in Tai Chi will exhibit higher levels of complexity across all measured physiological systems;*  *H.1.b) Those who participate in Tai Chi will exhibit higher levels of physical and cognitive function;*  *H.1.c) Those who participate in Tai Chi will exhibit higher levels of adaptive capacity;*  *H.1.d) The magnitude of the effects of Tai Chi on physiological complexity, function and adaptive capacity in healthy older adults will be positively correlated with age.*  **Specific Aim #2: To determine the relationships between biomarkers of physiological complexity, conventional measures of function and adaptive capacity.** Statistical regression models will be used to determine relationships, both at baseline and overtime, between a) complexity biomarkers and measures of physical and cognitive function, and b) complexity biomarkers and adaptive capacity. Elucidating these relationships will further inform the interpretation of complexity biomarkers and provide insights into underlying component mechanisms contributing to complex physiological dynamics.  ***Hypotheses*** *At baseline:*  *H.2.a) Measures of complexity are positively correlated with physical and cognitive function;*  *H.2.b) Measures of complexity are positively correlated with adaptive capacity.*  *Over time:*  *H.2.c) Changes in complexity are positively correlated with changes in physical and cognitive function;*  *H.2.d) Changes in complexity are positively correlated with changes in adaptive capacity.*  **Specific Aim #3: To determine cross-sectionally if long-term Tai Chi training (> 5 years) is associated with greater complexity, function, and adaptive capacity of multiple physiological systems in older healthy adults, when compared with Tai Chi naïve and novice (≤ 6 months training) Tai Chi practitioners**  ***Hypotheses*** *Compared to Tai Chi naïve and novice Tai Chi practitioners:*  *H.3.a) Long-term Tai Chi practitioners will exhibit higher levels of complexity across all measured physiological systems;*  *H.3.b) Long-term Tai Chi practitioners will exhibit higher levels of physical and cognitive function;*  *H.3.c) Long-term Tai Chi practitioners will exhibit higher levels of adaptive capacity;*  *H.3.d) The magnitude of the effects of long-term Tai Chi training on physiological complexity, function and adaptive capacity in healthy older adults will be positively correlated with age.*  **Specific Aim #4: To determine cross-sectionally if age is associated with greater complexity, function, and adaptive capacity of balance and gait in young healthy adults, when compared with to older adults**  ***Hypotheses*** *Compared to older adults and a young adult comparison group:*  *H.3.a); The magnitude of physiological complexity, function and adaptive capacity will be positively correlated with age.*  *H.3.b) Young adults will exhibit higher levels of adaptive capacity of balance and gait.* |
| --- |

#### B2. SIGNIFICANCE AND BACKGROUND FOR THE STUDY

| **Aging and the need for translational research**  Understanding the biology of aging and age-related disease is a growing priority in the U.S. By 2030, the U.S. population aged 65 and older will double to about 71 million older adults––i.e. one in every five Americans.^27^ This demographic shift is projected to increase U.S. health care spending by 25%. Accordingly, key components of the National Institute on Aging/NIH strategic plan include the development of biomarkers and innovative methodologies that improve our understanding of healthy aging and age-related disease, and the evaluation of novel interventions for the elderly. The use of complexity markers to evaluate Tai Chi and age-related physiological processes directly targets these initiatives.  **Complexity as a framework for understanding health and aging**  Healthy aging and physiologic function requires the complex integration of multiple control systems, feedback loops, and regulatory processes that enable an organism to adapt to the exigencies of everyday life. Control systems of the human body exist at molecular, cellular, organ, and systemic levels of organization, and operate over multiple time scales. To date, the predominant paradigm of medical research has been a reductionist one—i.e. dividing complex problems into smaller and simpler components and evaluating the relevance of these isolated components to physiological function and health.^28,29^ This ‘bottom-up’ approach has and continues to be extremely informative. However, there is a growing appreciation that a more ‘top down’, holistic/integrated approach to medical research and care is necessary to complement the predominant reductionist approach.^28,29^ Aging, itself, is a complex, systemic phenomenon that manifests from concomitant deterioration of multiple molecular, cellular, and organ systems. The progressive impairment in physiologic control systems and regulatory processes leads to diminished physical and cognitive function and to a decreased ability to adapt to stress.^3,4,30^ Due to its capacity to characterize complex dynamics within and between physiological systems, the emerging field of complex systems biology and its array of quantitative tools, show great promise for improving our understanding of aging, monitoring senescence, and for evaluating novel interventions, including complementary and alternative therapies, that treat age-related disease and promote healthy aging.^7,8^  **Measurement of complexity**  Generally speaking, complex signals typically exhibit one (and usually more) of the following properties: (i) non-linearity–the relationships among components are not additive; therefore small perturbations can cause large effects; (ii) non-stationarity–the statistical properties of the system’s output change with time; (iii) time irreversibility or asymmetry–systems dissipate energy as they operate far-from-equilibrium; (iv) multiscale variability–systems exhibit spatio-temporal patterns over a range of scales.^8^  Regardless of the underlying mechanisms of non-linear dynamics in physiologic systems, a robust body of research has demonstrated that indices of complexity can serve as sensitive and meaningful biomarkers. For example, complexity measures related to HR dynamics are stronger predictors of mortality in the elderly and post-myocardial infarction^9,10^ than linear spectral measures of heart rate variability. Non-linear measures of HR dynamics also: (1) are independent predictors of survival in patients with depressed left ventricular function^37^ and heart failure^38,39^; (2) are predictors of vulnerability to life threatening arrhythmia^40^; (3) can distinguish subjects with coronary artery disease from healthy controls^41^; and (4) can identify frailty in elderly populations.^42^ Similarly, complexity measures of center of pressure dynamics have been shown to discriminate athletes with head traumas better than conventional balance and function tests,^43,44^ and measures of gait dynamics more effectively distinguish fallers from non-fallers^45,46^ and provide helpful markers for characterizing neuromuscular disorders such as Huntington’s, ALS, and Parkinson’s disease.^47,48^  A number of techniques have been developed to quantify the dynamics of complex biological signals. Approaches derived from the fields of nonlinear dynamics and statistical physics include those based on the concepts of fractals and entropy. Generally speaking, indices of complexity differ from the conventional linear methods because the former considers the dynamics of the output; i.e., the moment-to-moment quality, scaling, and correlation properties of signals.^13^  **Aging and loss of complexity**  Aging is traditionally associated with the process of decline in physiological function.^55^ A central premise of this application, initially hypothesized by members of our team,^3,56^ is that physiological aging results from gradual changes in the underlying mechanisms of physiological control (i.e. regulatory networks of nonlinear feedback loops), leading to changes in physiological dynamics. Under this hypothesis even healthy subjects exhibit breakdown of certain feedback loops acting at different time scales in the regulation of physiological systems. This breakdown leads to a loss of physiological complexity. Below we summarize studies supporting this hypothesis, focusing on age-related loss in complexity in cardiovascular, locomotor, and balance systems.  Age-related loss of complexity in cardiovascular systems  Several studies have shown loss of heart rate (HR) complexity with aging.^12,13,56,57^ Pikkujammsa^12^ quantified variation in heart rate over 24 hrs using fractal and entropy-based measures. Participants included 114 healthy subjects ranging from 1 to 82 years old. When data were analyzed using broad age groups, children (<15 years old) and young adults (<40 years old) showed similar high levels of HR complexity. However, in older individuals, i.e. 40-82 years old, average complexity was lower and a statistically significant and progressive loss of complexity with age was observed. Using similar complexity indices, Beckers and colleagues^13^ evaluated HR dynamics of a total of 276 men and women between the ages of 18 and 71. Complexity indices were negatively correlated with age above, but not below the age 40y. As discussed above, loss of complexity in HR dynamics has been associated with cardiovascular disease and risks.  Age-related loss of complexity in locomotion and balance  *Gait dynamics and aging*: In one of the first studies to evaluate fractal properties of gait dynamics, members of our team evaluated fluctuations in the duration of stride cycles of healthy young men.^63^ Detrended fluctuation analysis (DFA) confirmed that stride-to-stride variation clearly exhibited long-range self-similar correlations extending over hundreds of steps. Subsequent studies have demonstrated that fractal-like gait fluctuations are present over longer intervals, at different speeds, and even when participants run or walk on treadmills.^64-66^  To test the hypothesis that quantitative measures of gait dynamics might be altered in elderly fallers, Hausdorff ^11^ compared gait dynamics in ambulatory community-dwelling older adults who were assessed at baseline and then followed for 12 months, monitoring fall status weekly. During the follow-up period, 20 of the 52 subjects reported a fall. Those who fell had reduced long-range fractal correlations in stride intervals. In contrast, measures of average gait speed and muscle strength did not differ between fallers and non-fallers. Other cross sectional studies also suggest that measures of gait dynamics and variability discriminate fallers from non-fallers^45,46^, and can characterize neuromuscular disorders such as Huntington’s, Parkinson’s disease, and ALS.^16,47,48^ These findings highlight the potential clinical utility of complexity-related measures of gait dynamics and their ability to provide quantitative measures that effectively characterize gait health and fall risk.  *Balance dynamics and aging:* A number of studies have showed that under resting conditions, COP time series exhibit complex variability over a broad range of time scales, and that that this complexity declines with age.^15,62,67,68^ For example, Costa and other members of our team^15^ evaluated short-term COP dynamics in 15 healthy young adults (avg. 27y), 22 healthy elderly adults (avg. 75y), and 22 elderly fallers (avg. 74y). Ten 30 second trials of quiet standing were used to capture COP velocity and displacement in both the antero-posterior (AP) and mediolateral (ML) planes. These trials were analyzed to calculate entropy-based complexity indices using multiscale entropy (MSE). For velocity measures, complexity indices in both the AP and ML plane were significantly different for all three groups; highest in young healthy and lowest in elderly fallers. Parallel trends were observed for COP displacement, but only differences in the AP plane were statistically significant. Similarly, Duarte and Sternad^62^ compared fractal (DFA) and entropy measures (MSE) of COP dynamics in 14 older (avg. 68y) and 14 younger (avg. 28) adults during a short (60 sec) and long (30 min) quiet standing task. Fractal-based measures suggested that complexity was lower in older subjects, but age-related trends were less clear using entropy measures, and were contingent on the duration of the standing period.  Members of our team recently evaluated complexity in COP dynamics from 551 participants (avg. 77.9y) whose frailty phenotype (not frail, pre-frail, or frail) was determined using standard criteria.^142^ Complexity of COP dynamics was quantified using MSE. Compared to the non-frail, MSE of COP was lower in pre-frail and frail groups (p<0.002). Although traditional linear measures of balance (root mean square amplitude of sway) were also associated with frailty, statistical models indicated that only MSE independently predicted frailty status after accounting for other physiologic determinants of balance such as age, vision, lower extremity strength, peripheral neuropathy, and frontal-executive cognitive function. MSE apparently quantifies an aspect of frailty not captured by other measures.  **Complexity and adaptability**  An important premise underlying this application is that the complexity of physiologic control systems serves an important purpose; it enables the organism to mount a focused adaptive response in order to perform a specific task or overcome an external stress. Therefore, baseline system complexity should predict one’s ability to adapt. The evidence supporting this hypothesis is still limited.  *Complexity and cardiovascular adaptability*: Blood pressure dynamics during standing provide one example of reduced adaptive capacity associated with age-related reduced complexity. In the supine resting state, BP oscillations produce a broad-band frequency spectrum with fractal (1/f) properties.^56^ When a healthy young subject is tilted upright to mimic standing, the BP frequency spectrum becomes less complex, giving rise to low frequency “Mayer waves” with a dominant frequency at about 0.1 H.^69^ This low frequency BP oscillation is thought to result from baroreflex-mediated vasomotor activity that counters the reduction in venous return to the heart through sympathetic activation and vasoconstriction, and prevents BP overshoots through sympathetic withdrawal and vasodilation. This feedback mechanism keeps BP in the range necessary to ensure adequate organ perfusion. Consistent with our hypothesis, elderly subjects have less complexity in BP dynamics in the supine position,^56^ and often fail to develop baroreflex-mediated systolic BP oscillations when tilted upright.^70^ This impairment may be associated with orthostatic hypotension or vasodepressor syncope.  *Complexity and responses to dual tasks during balance*: During the past two decades, increasing evidence suggests that healthy balance and gait are not simply automated motor functions, but functions that require higher-level cognitive input.^71,72^ Consequently, dual task studies that require a subject to split their attention between cognitive functions (e.g. simple arithmetic tasks) and motor control provide a convenient perturbation paradigm for studying gait and balance.^73^  In the study described above evaluating COP dynamics as a marker for frailty,^142^ our team also explored the effects of a dual cognitive task (counting backwards by 3’s from 500) on COP dynamics. Complexity of COP dynamics decreased significantly with dual tasking for all three groups evaluated (non-frail, pre-frail, frail). While not statistically significant, the magnitude of loss of COP complexity was lowest in the group with the highest baseline complexity (non-frail; 27% reduction) and greatest in the group with the lowest baseline complexity (frail; 81% reduction). This study suggests that cognitive tasks are valid perturbations for evaluating adaptive capacity to balance, and that loss of complexity may limit responsiveness to perturbations.  *Our proposed study will contribute significantly to further evaluating the hypothesis that complexity during basal conditions improves the ability of physiological systems to adapt to perturbations. Importantly, we propose to evaluate complexity and adaptive capacity of three different physiologic systems (cardiovascular, balance, and locomotor) within the same cohort of subjects, thus allowing us to compare/contrast complexity-related characteristics of different systems, and how they vary with Tai Chi training*.  **Restoration of complexity**  Only a limited number of studies have evaluated whether age- or disease-related loss of complexity can be restored to physiologic systems. Tulppo et al^74^ demonstrated that 8-weeks of a moderate- and high-volume aerobic exercise training program in sedentary healthy young adults increased fractal-like correlations in HR data. Heffernan et al^75^ demonstrated that 6 weeks of resistance training in healthy young men improves fractal properties of HR dynamics. Finally, members of our team demonstrated that subsensory mechanical noise applied to the feet via vibrating insoles can restore COP complexity in elderly subjects and those with diabetic neuropathy and stroke.^15,76^  *Given the limited research evaluating if age-related loss of complexity can be restored, testing the hypothesis that Tai Chi training is associated with improved complexity in multiple physiologic systems will represent a significant contribution to the field of aging research.*  **Tai Chi**  Tai Chi, also referred to as Taiji, Tai Chi Chuan or Taijiquan, is a mind-body exercise that originated in China, and that is growing in popularity in the West. Tai Chi is based on slow intentional movements, often coordinated with breathing and imagery, that aims to strengthen and relax the physical body and mind, enhance the natural flow of ‘qi’ (or life energy), and improve health, personal development, and in some systems, self defense.^77^ Recent surveys suggest that approximately 5 million Americans have practiced Tai Chi, and this number is increasing.^78,79^ Tai Chi shows great potential for becoming widely integrated into initiatives related to healthy aging. Tai Chi appears to be safe, even for the elderly and deconditioned, and clinical and community based studies report high adherence and enjoyment. Moreover, Tai Chi may be relatively cost-effective, requiring no special equipment or facilities. For these reasons, a growing body of clinical research has begun to evaluate the efficacy of Tai Chi as a therapy for a variety of age-related health issues; this research has been critically evaluated in recent reviews.^21,22,80-84^  3.8.1 Research evidence  *Cardiovascular systems*: Numerous studies suggest Tai Chi may positively impact cardiovascular health. Both cross-sectional and longitudinal studies indicate that Tai Chi training is associated with improved cardiorespiratory fitness, aerobic capacity and functional status.^85-88^ Randomized trials in cardiovascular populations have reported that Tai Chi favorably affects symptoms of heart failure,^20,90^ coronary artery disease,^91^ and recovery from coronary bypass surgery and infarction.^92,93^ However, studies evaluating the effects of Tai Chi exercise on HR dynamics are very limited. In one small study, elderly adults were randomized to 12 weeks Tai Chi vs. brisk walking.^95^ Following the intervention, linear frequency domain analyses showed trends towards increases in high frequency power and decreased in low frequency power in the Tai Chi group, but not the walking group. Both trends were interpreted as improvements in autonomic balance. A few other studies have evaluated the acute effects of Tai Chi on heart rate variability.^96,97^ However, we are not aware of any published studies evaluating the impact of Tai Chi on non-linear measures of HR dynamics. Our proposed research will add significantly to our understanding of how Tai Chi impacts cardiovascular health and aging as reflected by biomarkers of HR complexity.  Balance system: The positive effects of Tai Chi on balance and postural control have been summarized in a number of systematic reviews.^21,98,99^ For example, we conducted a systematic review of Tai Chi’s effects on balance.^100^ Of the 24 studies that met our inclusion criteria, 18 (including 8 RCTs) showed evidence that Tai Chi improves outcomes related to balance (e.g. greater single leg balance times, lower extremity strength and flexibility, and reduced fear of falling). In contrast, the benefits of Tai Chi for reducing falls during daily activities is less clear.^101^ While a number of large, sound RCTs have reported that Tai Chi significantly reduces fall risk in older adults,^102-107^ other studies have reported only modest^108,109^ or no effects.^110-112^ Tai Chi’s impact on postural sway is similarly ambiguous. While some studies have reported that Tai Chi reduces linear measures of sway (i.e, average sway area or velocity),^113-116^ these improvements are generally only observed under challenging conditions (e.g. standing with absent or conflicting sensory feedback).^117-119^ A hallmark study by Wolf and colleagues^106^ compared the effect of 15 weeks of computerized balance training vs. Tai Chi on postural sway and fall risk in older adults. Balance training, but not Tai Chi, reduced the average magnitude and velocity of sway. Under certain conditions, average sway magnitude was actually greater following Tai Chi. Interestingly, however, the risk of falling was significantly reduced only in the Tai Chi group (adjusted RR 0.51 vs. 0.98 for Tai Chi and balance training, respectively). Wolf and colleagues^105^ suggested that Tai Chi-related changes in fall risk may be due to factors other than average sway characteristics. They also suggested that the inherent aim of Tai Chi training might not be to reduce sway, but rather to provide corrective skills and improve confidence for managing postural instability. These results intrinsically suggest that the non-linear measures we propose in this application, which focus on quantifying the moment-to-moment control of postural sway, may afford unique insight into Tai Chi’s effect on balance and mechanisms of postural control.  *Locomotor system:* Relatively few studies have examined the effects of Tai Chi on long range gait dynamics. However, research combining various motion analysis systems and electromyography has begun to characterize unique properties of Tai Chi movements that may contribute to healthy gait. For example, descriptive studies of Tai Chi practitioners have reported that compared to normal gait, lower extremity movements during Tai Chi have: longer cycle duration and longer duration of single-leg stance time; greater ankle, knee, and hip joint motion; a larger lateral body shift; distinct plantar pressure distributions; and greater and unique patterns of lower extremity muscle activation.^23,120-124^ In a longitudinal study of frail elders, Hass et al^125^ reported that 48 weeks of Tai Chi training improved neuromuscular coordination and the mechanism by which forward momentum is generated during gait initiation. Gatts and Woollacott evaluated the effect of simulated slips on balance-impaired older adults following musculoskeletal surgery.^126^ Compared to a standard care control group, Tai Chi training reduced instability following slips; i.e. gait showed greater center of gravity movement without destabilization. A companion study also revealed faster reaction times of lower extremity muscles (assessed with electromyography) in the Tai Chi group.^127^ Faster neuromuscular reaction times following perturbations have also been reported in cross sectional studies comparing long-term Tai Chi practitioners to healthy sedentary controls or practitioners of other sports.^128-131^  These studies suggest that Tai Chi may improve certain gait characteristics and the capacity of the locomotor system to respond and adapt to physical stresses. However, nearly all of the above studies were based on average stride characteristics, with averages typically derived from multiple trials of very few gait cycles (e.g. 2-3 steps!). Surprisingly, few Tai Chi studies have assessed gait dynamics over longer distances/times^94,109-111,132^ In a few studies which have assessed gait over relatively longer periods of time, improvements in standard measures such as average gait speed are often not correlated with fall risk, balance and other functional measures.^109,133,134^ Our proposed study will advance our understanding of Tai Chi’s impact on the locomotor system by employing indices of complexity to accurately characterize long-term inter-stride dynamics, as well as the ability of the locomotor system to adapt to stress induced by cognitive dual tasking. |
| --- |

#### B3. DESCRIPTION OF RESEARCH PROTOCOL

##### Study Design – Overview, Methods, Procedures

| We propose a two-arm prospective randomized clinical trial with non-randomized third group to evaluate the effect of Tai Chi on age-related loss of complexity.    **Arms 1 and 2**  will consist of a total of 60 Tai Chi–naïve healthy older adults (aged 50-79) will be randomized to either six months of Tai Chi training, or to a waitlist control receiving standard medical care. Our primary outcomes are complexity-based measures of cardiovascular (heart rate, HR), balance (center of pressure, COP) and locomotor (stride interval) dynamics assessed at 3 and 6 months. Secondary outcomes include measures of physical and psychological function and tests of physiological adaptability also assessed at 3 and 6 months. A secondary goal of this study is to characterize the relationship between complexity biomarkers, measures of function, and adaptability. This pilot study will inform a future more definitive trial by providing information on recruitment and retention, compliance, dose-dependent effects, preliminary estimates of effect size, and the optimal biomarkers of complexity, function, and adaptive capacity. Figure 1 summarizes the overall study design.  **Arm 3** (Tai Chi Expert Group): A total of 30 healthy older adults (aged 50-79) each with over 5 years of Tai Chi practice will comprise the third group of this study. In a single visit, primary outcomes related to complexity-based measures of cardiovascular (heart rate, HR), balance (center of pressure, COP) and locomotor (stride interval) dynamics), as well as secondary outcomes related to physical and psychological function and tests of physiological adaptability will be assessed. This group of Tai Chi experts will serve as a ‘gold standard’ of what the long-term physiological impacts of tai chi training might provide, and thus serve as an informative reference population to our current Tai Chi Naïve and Tai Chi Novice (i.e. after 6 months training) groups.  **Arm 4** (Young Comparison Group): A total of 15 healthy young adults (aged 25-35) with no prior Tai Chi experience will comprise the fourth group of this study. In a single visit, primary outcomes related to complexity-based measures of balance (center of pressure, COP) and locomotor (stride interval) dynamics, as well as secondary outcomes related to physical function and physiological adaptability will be assessed. This group of healthy young adults will serve as a reference comparison group for balance and gait measures before any age-related decline begins.  **Tai Chi intervention**  All Tai Chi instruction will take place at one of the Tai Chi schools within our research network, which was established and used in a prior NIH funded Tai Chi trial of ostepenic women (BIDMC protocol # 2007P––000162). These schools were chosen to meet specific criteria ensuring they provide valid, stable Tai Chi programs led by experienced teachers. Importantly, since participants are being asked to make a six month commitment to Tai Chi training, allowing them to receive training at a school they choose within their community will increase the likelihood of protocol adherence. Administering the intervention through community Tai Chi schools also affords a high level of ecological validity, as participants will be exposed to a number of traditional components of Tai Chi training that are often absent in fixed protocols provided in medical settings.^77,152^ Finally, because of the inclusion of multiple Tai Chi instructors and well-defined but slightly varying protocols, the results of this study will have good generalizability—applying not only to a single protocol of Tai Chi taught by one teacher, but to a range of approaches that share a common, well-defined set of criteria.  Participants randomized to Tai Chi will be required to attend, on average, two classes per week over the six months of the intervention. They will also be asked to practice a minimum of 30 minutes, two additional days per week. The majority of schools in our network provide DVD’s or printed materials to facilitate home practice. Compliance with this training schedule will be monitored using a combination of class attendance cards that are signed by Tai Chi instructors following each visit, and participant-completed home practice logs employing protocols developed in our current Tai Chi for osteopenia study. Participants will be asked to send class attendance and home practice logs to study staff monthly using prepaid envelopes provided at the beginning of the study. If attendance and/or practice logs are not submitted on time, the study coordinator will call the participant within 7 days of the due date. Effort will be made to keep subjects actively engaged in the protocol. If a participant is non-compliant, the study coordinator will contact the participant and work with them to overcome any barriers to participation.  **Measurement protocols**  Overview of measurement protocol  All three arms of the study will complete our baseline measurement protocol including acquisition of 1) steady-state dynamics of the cardiovascular, locomotor and balance control systems, 2) measures of the ability of these systems to respond to imposed stressors, and 3) more traditional measures of function and Health Related Quality of Life (HRQOL). Additionally, traditional linear outcome measures for heart rate variability, gait, and balance will be assessed for comparison with complexity measures. All measurements will be assessed at baseline. Only Arms 1 and 2 (Tai Chi Novices and Control Group) will repeat all measures at 3 and 6-months. All outcome assessors will remain blinded with respect to treatment allocation. Table 1 provides a summary of these measures, as well as additional screening tools.  **Table 1. Summary of outcomes and measures and associated variables (see below)**   \| **Temporal dynamics during steady-state conditions** \| **Physiological measure** \| **Testing methods** \| **Outcome variable** \| \| --- \| --- \| --- \| --- \| \|  \| Heart rate (HR)º \| Beat-to-beat variation measured using ECG for a 30 minute during seated quiet resting \| HR complexity \| \|  \| Standing balance \| Center of pressure (COP) dynamics during quiet standing with eyes open \| COP complexity \| \|  \| Gait \| Stride–to-stride interval at preferred speed during a 20 minute period \| Stride complexity \| \| **Adaptive capacity** \| Heart rateº \| Sit to stand test \| Max change in heart rate \| \|  \| Standing balance \| Eyes closed  Cognitive dual task \| Change in COP complexity  Change in magnitude of COP  displacement \| \|  \| Gait \| Cognitive dual task \| Change in average stride  variability \| \| **Function and HRQOL** \| Exercise capacity \| Max walking speed \| m/sec \| \|  \| Musculoskeletal strength/power \| a. Lower extremity power (Jump test)  b. Grip strength (dynamometer) \| a. Maximum height of  jump (cm)  b. kg \| \|  \| Timed Up and Go (TUG) \| Standardized test of time to rise from chair, walk 10 feet to and from cone, return to seated position \| Functional status \| \|  \| Functional reach \| Subject reaches forward with hand and arm  extended and parallel to a yardstick at  shoulder height to reach as far as possible without taking a step \| Functional Status \| \|  \| Clinical balanceº \| Single leg balance with eyes open and closed \| Maximum time on balance \| \|  \| Range of motionº \| a. Goniometry of lower extremity (hips, knees, ankles bilaterally)  b. Sit and reach test (back and leg flexibility) \| a. Degrees of motion  b. cm \| \|  \| Respiration rateº \| Nasal thermoster attached to volume gas monitor \| Breathing frequency \| \|  \| Electrical potential \| Skin surface electrical potential \| Skin surface electrical potential at select segments along arms and legs \| \|  \| Moodº \| Profile of Mood States \| Indices of 6 domains of mood \| \|  \| Cognitive functionº \| Trail Making Test A and B  Controlled Oral Word Association Test  Backwards Digit Span Test \| Time to complete test (sec)  Number of words recalled/min  Number of digit sequences  correctly repeated \| \|  \| HRQOLº \| SF-36 \| Indices of physical and  mental health in 8 domains \| \|  \| Physical activityº \| Physical Activity Status Scale  Arm 3 ONLY: Frequency of Tai Chi and related exercise training \| Index of physical activity  during prior week \| \| **Protocol adherence, screening, and potential confounders** \| Tai Chi training*º \| Teacher reported class attendance records  Self-reported home practice logs \| Numbers of classes/month  Hours of home practice/month \| \|  \| Cognitive function \| Mini-mental state exam \| Index reflecting 5 domains of  cognitive health \| \|  \| Expectancy regarding intervention*º \| Expectancy/credibility questionnaire \| Numerical scale (1-5) \| \|  \| Satisfaction regarding intervention*º \| Satisfaction questionnaire \| Numerical scale (1-5) \| \|  \| Exit Interview*º \| Qualitative exit questions concerning study experience. \| Six open-ended questions \|   (Key provided on following page)  * Arm 3 (The Tai Chi Expert Group) will not be subjected to these measures.  º Arm 4 (Young Comparison Group) will not be subjected to these measures.  Flow of participant through protocol measurements (Arm 1,2, and 3)  All testing will take place in the Syncope and Falls in the Elderly (SAFE) laboratory, located within the CRC at BIDMC. Subjects will be asked to arrive at the laboratory between 8:00 -10:00 a.m. We expect testing to occur for approximately 4 hours. Participants will first meet with a CRC study nurse who will confirm eligibility questions asked during the phone screen. A study investigator will then ask participants to review and sign the informed consent form. The CRC study nurse will then review participant’s health history, obtain vital signs, ECG, height and weight. Study personnel will implement the mini-mental state exam. Participants that meet all eligibility criteria will then undergo standard study related measurements that will be conducted by Dr. Manor and/or a TBD research assistant, with oversight by Dr. Novak, the SAFE laboratory director.  The sequence of testing we will employ is based on protocols regularly conducted in the SAFE laboratory,^140,153^ and has been designed to minimize fatigue and patient burden, and maximize the quality/validity of the data collected. Each stage of testing, as well as repeated assessments of specific tests, will be separated by an appropriate resting period. The total time for completion of measurements is estimated to be 4 hours. The general order of procedures is as follows:  a. Fitting of HR instrumentation, measurement of resting HR dynamics, sit-to-stand test, and timed up and go test  b. Steady state and challenged center of pressure (balance) testing  c. Preferred speed stride dynamics and dual task walking  d. Break with snack  e. Completions of cognitive, mood, HRQOL, and exercise behavior instruments  f. Physical function tests (maximum walking speed, balance, jumping height, grip strength, range of motion)  Flow of participant through protocol measurements (Arm 4 – Young Comparison Group)  All testing will take place in the Syncope and Falls in the Elderly (SAFE) laboratory, located within the CRC at BIDMC. We expect testing to occur for approximately 2 hours. Participants will first meet with a CRC study nurse who will confirm eligibility questions asked during the phone screen. A study investigator will then ask participants to review and sign the informed consent form. The CRC study nurse will then review participant’s health history, obtain vital signs, height and weight. Study personnel will implement the mini-mental state exam. Participants that meet all eligibility criteria will then undergo standard study related measurements that will be conducted by Dr. Manor and/or a TBD research assistant, with oversight by Dr. Novak, the SAFE laboratory director.  The sequence of testing we will employ is based on protocols regularly conducted in the SAFE laboratory,^140,153^ and has been designed to minimize fatigue and patient burden, and maximize the quality/validity of the data collected. Each stage of testing, as well as repeated assessments of specific tests will be separated by an appropriate resting period. The total time for completion of measurements is estimated to be 2 hours. The general order of procedures is as follows:  a. Timed up and go test  b. Steady state and challenged center of pressure (balance) testing  c. Preferred speed stride dynamics and dual task walking  d. Physical function tests (maximum walking speed, balance, jumping height, grip strength, range of motion)  Details of measurement procedures  *Cardiovascular measures:* Steady-state HR dynamics will be assessed during seated rest with spontaneous breathing. To collect the sufficient number of heartbeat cycles for complexity measures, HR will be assessed for 30 minutes. Additionally, two 5-minute periods of HR dynamics will also be assessed during paced breathing (15 and 6 breaths per minute); paced breathing will enable the assessment of cardiovascular dynamics and linear measures of HR variability while controlling for the potentially confounding effects of respiration.^154^ During each breathing pattern, HR and respiration patterns will be simultaneously measured. Heart rate will be recorded using a 3-lead electrocardiogram attached to a light-weight (<300 g) wearable ME6000 data acquisition computer (Mega Elektronika, Inc). Respiration will be measured using a nasal thermistor attached to an infrared end-tidal volume gas monitor (Capnomac Ultima, Ohmeda Inc.). Beat-to-beat blood pressure will be measured from a cuff placed on the finger using a Portapress-2 device (FMS, Inc), and galvanic skin conductance will be measured using an AD Instrument MLT116F (AD Instrument, Colorado Springs, CO).  The effects of perturbation to the cardiovascular system will be evaluated from the well-validated sit-to-stand test.^155^ Standing following prolonged sitting (≥5 min) results in an immediate elevation in HR, with relative greater change in HR response reflective of impaired cardiovascular regulation. The maximum change in HR (ΔHR) evoked from the sit-to-stand test will be quantified. ΔHR will be calculated by subtracting the minimum HR during sitting from the maximum HR during the subsequent bout of 1 min standing.  *Balance measures:* Steady-state standing balance dynamics will be assessed during eyes-open quiet standing as well as sitting. With arms at the side and bare feet shoulder width apart, subjects will be asked to stand as still as possible and to visually fixate on an “X” drawn on a wall approximately 3m away at eye-level. Center of pressure (COP) displacement will be recorded with a standard force plate (Kistler Instruments Corp, Amherst, NY). To collect sufficient COP data for complexity analysis, 1 min of continuous standing will be completed. This protocol will repeat for the sitting component of the test, with modifications made for the now seated subject.  The effects of visual and cognitive perturbations will also be evaluated. For the visual perturbation, subjects will stand similar to above, except with eyes-closed.^58^ For the cognitive perturbation, subjects will perform the dual task of counting backwards, out loud, in multiples of three, while attempting to repeat the steady-state protocol. Subjects will wear an MP3 recording device attached to their person or nearby to record verbal dual task cognitive test performance. This cognitive dual-task is commonly used to challenge standing balance in both healthy and movement disordered populations.^72^ To avoid potential effects of learning, subjects will begin each trial from 500, 499, or 498, in random order.  To account for potential trial-to-trial variability, three 1 min trials will be completed for each condition (steady-state, eyes-closed, dual task). Trials will be randomized and at least one minute rest will be given between each trial. During the first trial, the subject’s feet will be outlined with chalk to ensure consistent foot placement. A trained spotter will provide stabilizing assistance if necessary.  As one's balance is influenced by breathing, the effects of breathing frequency on standing balance will also be examined during one 4 min trial of standing on the force plate. Following 30sec of normal breathing, subjects will be asked to breath in rhythm to a metronome for three minutes. Metronome frequency will change every 30sec to obtain the following breathing frequencies: 50, 40, 30, 20, 10 and 5 breaths/min. A final 30sec of normal breathing will be completed to finish the test. The subject will be given time to practice breathing in rhythm to the metronome prior to the test. They will be reminded to return to their normal breathing patterns should they feel uncomfortable or light-headed during the test, and encouraged to sit or rest at any time. A trained spotter will remain behind the subject at all times.  *Gait measures:* Steady-state gait dynamics will be assessed during over ground walking at preferred speed. Subjects will walk along a long corridor (~75m) that is wide enough (~5m) to enable smooth turning. To record consecutive stride-to-stride durations, subjects will have foot-switches (Mega Elektronika, Inc) inserted into the shoes and the ME6000 data acquisition monitor attached to the waist. To collect the sufficient number of steps required for complexity measures, 20 min of continuous walking will be completed.The effects of a locomotor perturbation on gait speed, an indicator of gait health and overall function,^156^ will be assessed during continuous over ground walking while performing the same cognitive dual task as in balance testing. Subjects will complete three 1 min trials while counting backwards, out loud, in multiples of three.^157^ Dual tasking decreases walking speed.^72^ The distance walked during each trial will be recorded and used to calculate average speed. *Assessment of function and health-related quality of life*  Multiple clinical tests of physical and mental function will further characterize the sample, evaluate correlations between complexity measures and function, and assess the impact of Tai Chi and age on these outcomes.  *Maximum Walking Speed*: Performance in this common, easily administered clinical test is associated with functional decline.^158^ Subjects will walk in a straight line as fast as possible, without running, on a pre-measured 11m course. The time taken to walk 5m, from the 3m to 8m mark will be recorded and used to calculate maximal walking speed (m/s).  *One-legged standing balance*: This test will be assessed according to Vereeck et al.^159^ Timing will start when the subject has assumed the proper position on their preferred leg and verbally indicates readiness to begin the test. Timing will stop when the subject disengages from the starting position (i.e., the suspended foot touches the floor or the hands touch support surface) or when the 30 sec time limit is reached. The test will be conducted in the corner of a room, and the subject will be instructed to reach for the wall should they feel unstable. Three trials will be completed for eyes-open and eyes-closed conditions, and the greatest duration (sec) for each condition will be used for analysis. This test has been correlated with fall risk in older adults.^160^  *Lower-Extremity Muscular Power*: Maximal power output of the lower extremities will be estimated by assessing maximum vertical jump height (m) according to Bosco.^161^ In a study of more than 6000 women and men aged 40 to 85 years, Fujita et al^162^ determined this test to be safe for community-dwelling older adults, and that those with relatively low vertical jump scores had higher mortality rate over the six year follow-up period. Furthermore, as compared to lower-extremity muscular strength, power appears to be closely related to fall-risk^163^ and physical function.^164^ Subjects will complete a standard warm-up consisting of five sub-maximal jumps. Subjects will then complete three countermovement jumps on a stationary Kistler force plate (Kistler Instruments Corp, Amherst, NY). The “flight-time” for each jump, determined from the force plate reading, will be used to determine the maximum height reached by the subject’s center of mass using the Bosco method.^161^  *Range of Motion*: Bilateral hip, knee, and ankle joint passive range of motion (degrees) will be measured by a trained investigator (Dr. Manor) using a goniometer and standard procedures^165^ Lower body flexibility will also be assessed using the validated Chair Sit and Reach test.^166^  *Electrical Potential:* Skin surface electrical potential will be measured at select segments along the arms and legs, using 8 surface band electrodes (EL-506, Biopac, Goleta, CA) – 2 electrodes per extremity.  The electrodes will be connected to a data acquisition unit (Powerlab ML-880, AD Instruments, Colorado Springs, CO). This procedure tests the hypotheses exploring relationships between electrical potential and dynamics of other physiological measures (e.g. blood pressure and breathing).  *Physical Activity Levels:* In addition to Tai Chi training, general daily physical activity levels may influence both physical and cognitive function. The amount and intensity will therefore be recorded using the Physical Activity Status Scale (PASS).^167^ The PASS scale is an extension of the four-category scale used by Jones and associates^168^ to quantify leisure exercise levels. Each subject will be asked to estimate his or her general physical activity during the previous week using an 11-point scale (i.e., 0-10). The scale quantifies physical activity duration by a combination of the minutes of exercise per week and the intensity of this exercise (heavy, modest, or none). The concurrent validity of the scale has been documented in both men and women and scores correlate with maximal oxygen consumption in younger and older adults.^169,170^  *Grip strength:* Maximum grip strength is widely considered a simple and reliable measure that is correlated with mortality/survival, and disability, and overall function in middle-aged and older adults.^171^ Grip strength will be measured using a handgrip dynanometer Grip D (Takei Scientific Instruments, Tokyo, Japan). Subjects will be asked to grip the instrument with their dominant hand with maximum force. Measurements will be recorded to the nearest 0.5 kg, repeated three times and averaged. *Timed Up and Go Test:* This is a validated test for quantifying functional mobility that may also be useful in following clinical change over time. Patients begin in a seated position, stand up from a chair, walk a short distance (3 meters, ~10 feet) at a comfortable pace, turn around, return, and sit down again. This test will be timed by the blinded research assistant. The patient will repeat the test and the times will be averaged. The test is quick, requires no special equipment or training, and is easily included as part of the routine medical examination. *Functional Reach:* This standardized test is a measure of balance and is the difference, in inches, between arm's length and maximal forward reach, using a fixed base of support (such as a wall). This test can be used to detect balance impairment, change in balance performance over time, and in the design of modified environments for impaired older persons. The test utilizes a 48-inch measuring device or "yardstick".A reach of less than or equal to a certain level could be predictive of fall risk.  *SF36*: This health-related QOL instrument consists of 36 multiple choice items scored on a 0 to 100 scale of improving health status for each of 8 domains: physical functioning, role limitation due to physical problems, bodily pain, general health, vitality, social functioning, role limitations due to personal or emotional problems, and mental health. The SF-36 has been validated in a variety of populations including young and old healthy individuals, and patients with chronic medical problems, and has good test-retest reliability.^172,173^  *Cognitive function*: Cognitive function will be assessed with validated measures of executive function, working and short-term memory. The Trail Making Test (TMT) is a widely used instrument that is administered in two parts.^174^ TMTa is a visual-scanning task; the time required to draw lines sequentially connecting numbered circles from 1 to 25 is recorded. TMTb assesses time required to connect the same number of circles in an alternating sequence of numbers and letters. TMTb is considered to evaluate executive control, and is correlated with other executive function measures.^175^ TMT is a sensitive indicator of overall neurological impairment,^174^ and has good reliability.^176^ The Controlled Oral Word Association Test (COWAT) examines working memory span.^177^ COWAT requires the participant to produce as many words as possible that begin with a given letter of the alphabet (F,A,S). There is 1 minute allowed for each of the three letters. The score is the sum of all acceptable words produced in the three trials. COWAT has good reliability and validity.^177-179^ The Backwards Digit Span Test (BDS) is a widely used measure of short-term memory, i.e. the number of digits a person can absorb and recall in correct serial order. Two trials of eight number sequences are read aloud. The number sequences start out easy, with just two numbers, and get progressively more difficult, the last being nine digits. Scores are based on the number of sequences correctly recalled (i.e., until the participant consecutively fails two trials of the same digit span length). BDS has good reliability and validity.^177^ All three measures have been employed in studies evaluating the impact of exercise on cognitive function in elders.^1,2^  *Mini-Mental State Examination (MMSE)*: This is a short assessment instrument used to grade cognitive function (orientation to time and place, registration, memory, attention and concentration, praxis, constructional and language capacity, ability to follow commands).^180^ It is used widely in primary care and in community based research settings.^181,182^ The inter-rater reliability of the MMSE is 0.83.^180^ MMSE will be used only as a screening tool; only participants with scores ≥24 will be included.  *Profile of Mood States (POMS):* This is a well-validated instrument for assessing emotional states that are transient and expected to respond to clinical intervention.^185^ It has been widely used to assess the effects of exercise interventions. The instrument consists of 65 single-word items rated on a 5-point scale to indicate recent mood in 6 dimensions: tension/anxiety, depression/dejection, anger/hostility, vigor/activity, fatigue/ inertia, and confusion/bewilderment. A decreased total score denotes an improved emotional state. Test-reliability coefficients are reported to range from 0.65 to 0.74.^185^ Studies of Tai Chi have reported improvement in mood, decrease in anxiety, and enhancement in vigor as measured by the POMS scale.^186^  *Expectancy and Satisfaction*: We will assess the degree to which participants believe that Tai Chi will be beneficial to their health using an instrument used in a number of prior CAM studies. Participants in both groups at baseline will be asked: ‘How confident are you that Tai Chi will improve your health?’; ‘How confident are you in recommending Tai Chi to a friend for health promotion?’; ‘Does doing Tai Chi for health make sense to you?’. Response choices range from 1 through 5, with 1 representing ‘not at all’ confident and 5 ‘extremely’ confident. For participants in the Tai Chi group, satisfaction with the intervention will be assessed by asking them to score the following four statements (5 point scale; 1 strongly agree, 5 strongly disagree) at 3 and 6 month visits: ‘Overall, I am satisfied with my Tai Chi experience in the study’; ‘Overall, I am satisfied with my Tai Chi school’; ‘Overall, I am satisfied with the tai chi teachers I am training with’; ‘I would recommend the Tai Chi program I am enrolled in to a friend or relative.’  *Qualitative Exit Interview:* At the end of your 6 month visit, subjects assigned to the Tai Chi group will also be asked to complete a qualitative exit questionnaire comprised of 6 questions. We will audio tape the answers to these questions. The audio file will be erased once it has been transcribed, within a year of the interview date. Subjects may decline any question they do not wish to answer, and they may decline from having their answers audio taped.  **Survey regarding control groups for future studies**  At the end of our initial phone screen, all participants will be asked a series of questions to explore the types of activities they would consider worthy of participating in, and willing to be randomized to, in a future hypothetical trial related to healthy aging. A list of specific activities (e.g., exercise classes at local YMCA’s, academic lecture series on healthy aging, and group walking classes) will be presented, and for each activity, participants will be asked to respond with one of the following assessments: very interested in participating, moderately interested in participating; not at all interested in participating. Results from this survey will help determine the controls we might employ in a follow-up multi-arm study.  **Data processing**  Analog signals from the ECG and balance platform will be recorded at 1000 Hz using Labview NIDAQ software (National Instruments Data Acquisition System 64 Channel/100 Ks/s, Labview 6i, Austin, TX). Foot switch signals will also be recorded at 1000 Hz using the ME6000 and accompanying software.  For cardiovascular measures, beat-to-beat HR will be determined from the R-wave on the ECG. All data will be visually inspected for accuracy of R-wave detection, artifacts and occasional extra-systoles using software written in our laboratory. Ectopy and artifacts will be removed using a linear interpolation algorithm. The HR interval time-series will be used to compute steady-state complexity measures. For comparison with complexity measures, HR data will also be analyzed using spectral analysis methods we have developed and employed in our prior studies.^137,144^  For balance measures, the acquired anterioposterior (AP) and mediolateral (ML) (COP) time series will be digitally filtered with a zero-lag fourth-order Butterworth low-pass filter with a 5 Hz cutoff frequency. AP and ML COP time-series will be used to compute steady state complexity measures (see D.6). For comparison with complexity measures, the traditional measures of average displacement (mm) in the AP and ML directions will also be determined by computing the mean scalar displacement along each axis.^189^ To assess the effects of visual and cognitive perturbation, average ML and AP displacement will also be calculated for the eyes-closed and dual task conditions. As each perturbation increases the magnitude of COP displacement, the effect of perturbation will be defined by the percent change in average ML and AP COP displacement during the perturbation condition as compared to the steady-state condition.  For gait measures, on/off foot switch data will be used to determine consecutive stride durations (time from initial contact of one foot to the subsequent contact of the same foot) during the 20 min walk. The resulting stride duration time series will be used to compute steady-state complexity measures. To assess the effects of the dual task perturbation (known to decrease walking speed), the percent change in walking speed during dual task as compared to the steady state condition will be computed.  **Computation of complexity measures**  Our team has extensive experience quantitatively analyzing complex physiologic signals – particularly for signals to be obtained in this study. Two principal complexity metrics––Detrended Fluctuation Analysis (DFA) and Multiscale Entropy (MSE)––were developed by team members.^6,26,51^ DFA quantifies long-range correlation properties (fractality) of a signal. Unlike alternative approaches (e.g., Fourier spectral analysis and Hurst analysis), DFA permits the detection of intrinsic self-similarity embedded in a seemingly nonstationary time series. DFA has been successfully applied to a wide range of simulated and physiologic time series in recent years.^5,13,18^ Multiscale Entropy quantifies the information content of a signal over multiple scales. Compared to other entropy-based methods, MSE uniquely accounts for dynamical information encoded in physiologic signals over multiple scales and therefore is able to distinguish between highly irregular random (uncorrelated) signals and truly complex ones.^52,190,191^ Extensive information on these measures, including details of algorithms, tutorials, and examples of their application is provided at Research Resource for Complex Physiologic Signals ([www.physionet.org](http://www.physionet.org)). This website is sponsored by NIH, and was developed by members of our team. |
| --- | --- | --- | --- | --- | --- | --- | --- | --- | --- | --- | --- | --- | --- | --- | --- | --- | --- | --- | --- | --- | --- | --- | --- | --- | --- | --- | --- | --- | --- | --- | --- | --- | --- | --- | --- | --- | --- | --- | --- | --- | --- | --- | --- | --- | --- | --- | --- | --- | --- | --- | --- | --- | --- | --- | --- | --- | --- | --- | --- | --- | --- | --- | --- | --- | --- | --- | --- | --- | --- | --- | --- | --- | --- | --- | --- | --- | --- | --- | --- | --- | --- | --- | --- | --- | --- | --- | --- | --- | --- | --- | --- | --- | --- | --- | --- | --- |

### Statistical Considerations

| ***Sample Size Justification:*** Our analysis will be based on a longitudinal regression analysis in which we do not have an estimate of the within-participant correlations. In the worst case, the two follow-up measures provide no more information than a single follow-up, and the power calculation is equivalent to that for a simple t-test. With 30 participants in each group, we would have 50% power to detect an effect size of 0.52 (i.e., the sample size provides 50% sensitivity to detect a difference between groups .52 times the standard deviation of the outcome measure) and 80% power to detect an effect size of 0.74 for each of the main effects. Our primary hypothesis, however, is an interaction effect. Our sample size provides at least 50% power to detect an interaction effect size of 1.03, under this worst-case scenario. At the other extreme, if the two follow-up observations are independent, we will have 50% power to detect an interaction effect size of 0.72 and 80% power to detect an effect size of 1.03. The use of regression models further increases power by accounting for additional sources of variability. In our heart failure study, the mean difference in MSE change between Tai Chi and control was 2.26, which translates to an effect size of 1.2. Preliminary COP data from our Tai Chi for osteopenia study showed a similar effect. Therefore, we think changes of this magnitude will likely be present in the proposed study and our study has adequate power.  For Study Aim 3, we will in essence be comparing the 30 tai chi experts to the 60 novices. After adjusting for the effect of age, we will have 80% power to detect a difference between naïve and tai chi experts of approximately 0.65 times the residual error.  For Study Aim 4, we will be comparing the baseline COP and balance measures in the various age categories (15 participants age 25-35, and 20 participants in each of the older groups). This sample will provide at least 82% power to detect a difference between the young group and the average of the older groups equal to the within-group standard deviation.  ***b. Data Analysis***  *Aim 1*: We are interested in comparing the change over time in the Tai Chi students versus the controls. The primary analysis will use an intention-to-treat paradigm, i.e., participants will be evaluated on the basis of group assigned by randomization without regard to subsequent adherence. Since this is a pilot study, we will not impute values for missing data; however, the statistical models we are using will include all available data. We recognize that some participants may drop out before the follow-up evaluation and that some outcome measures may not be evaluable for some participants. We will make no adjustment for multiple testing. A secondary ‘per-protocol’ analysis will be limited to participants who were compliant (attended 70% of classes and completed at least 70% of home sessions).  Our primary analysis will employ linear mixed effects regression models that examine change over time (i.e., slope) for each outcome measure (i.e., the complexity measures, MSE and detrended fluctuation analysis) for each of the systems (i.e., heart rate, balance and gait). The models will incorporate a random intercept and a random slope for each participant. The models will be of the form:  ****  for individual $i$ in treatment group $j$ where the random intercept $\alpha_{i}\sim N(0,\sigma_{\alpha}^{2})$, the random slope $\beta_{2i}\sim N(0,\sigma_{\beta_{2}}^{2})$, and the residual error $\varepsilon_{ij}\sim N(0,\sigma_{\varepsilon}^{2})$. The focus of the primary analyses is on $\beta_{3}$, which evaluates whether treatment is associated with the slope over time.  We will also conduct sensitivity analyses that incorporate additional covariates into the models, including age, gender, baseline physical and mental health, BMI, expectancy, and exercise behavior. We are particularly interested in examining age with a focus on assessing whether age substantially reduces the variability of the random effects, i.e., whether it explains a substantial proportion of between-person variability in baseline complexity and slope.  Analyses of secondary outcomes will follow the same general analytic approach. We will use mixed effects models to examine the effects of Tai Chi training over time on physical and cognitive function (exercise capacity, balance, upper and lower extremity strength, cognitive function, and quality of life) and adaptive capacity (change in heart rate, change in COP displacement, change in stride variability).  *Aim 2*: We hypothesize that function and adaptive capacity are associated with complexity. We will first examine the association between complexity measures and function/adaptive capacity at baseline. We will calculate Pearson correlation coefficients between the complexity measures (MSE and detrended fluctuation analysis) and the measures of function/adaptive capacity. To examine the independent association between complexity and function/adaptive capacity, we will fit ordinary least squares regression models using the function/adaptive capacity measures as the dependent variable. Independent variables will include age and sex as well as any other baseline characteristics associated with the function variable. We will add the complexity measure to this model and evaluate the Wald test and the change in R^2^.  We will also investigate whether changes in complexity are associated with changes in function/adaptive capacity. We will fit linear regression models with change in function/adaptive capacity as the dependent variable and change in complexity as the independent variable of interest. Since we will have 2 observations per participant (change at 3 months and change at 6 months), we will use generalized estimating equations methods (GEE) to account for the within-person correlation. Independent variables will include the measure of function/adaptive capacity at baseline, age, sex, treatment group and time (3 vs. 6 month).  Further, we hypothesize that complexity is the primary mediator of the association between Tai Chi and improvements in adaptive capacity. We will examine this using the approach recommended by Judd and Kenny.^193^ For example, to examine whether multi-scale entropy of balance mediates the effect of Tai Chi on center of pressure response to a dual cognitive task among the older participants, we will fit 3 regression models: regress MSE on Tai Chi use; regress COP response on Tai Chi; and regress COP response on MSE and Tai Chi use. The associations seen in these models determine the existence and strength of mediation.  Despite the implicit directionality of our hypotheses, all tests will be two-sided. We recognize that we are examining a moderate number of outcomes in this study. Since it is a pilot study and has limited power, we do not intend to adjust for multiple testing.  *Aim 3:* Our goal is to compare the complexity measures, function, and resilience to perturbations between tai chi experts and those with no tai chi training or only limited training. We expect responsiveness to decrease as a function of age among the tai chi naïve participants (i.e., both randomized groups combined). We hypothesize that tai chi experts will have a generally higher level of responsiveness, and that their responsiveness will decline with age at a slower rate than naïve or novice practitioners. To examine these questions, we will fit linear regression models to the baseline responsiveness measures for all participants: $y_{i}=\alpha+\beta_{1}TC+\beta_{2}(Age-50)+\beta_{3}[TC*(Age-50)]+\varepsilon_{i}$   where TC is an indicator variable for the tai chi experts. $\beta_{1}$ is an estimate of the difference in responsiveness between tai chi experts at age 50, $\beta_{2}$ is an estimate of the change in responsiveness per year for the novice group, and $\beta_{3}$is the difference in rate of change between novices and tai chi experts.  We will fit a similar model to compare the tai chi experts to the naïve and novice groups using the 6-month data for the randomized patients and the baseline data for the tai chi experts. The group variable will be a categorical variable with 3 levels.  *Aim 4:* We will calculate sample means for each of the outcomes in each age group. We will use standard one-way analysis of variance to compare the four groups. If the overall test is significant, we will use Dunnett’s test to compare the young group to each of the older age groups. |
| --- |

### C. Subject Selection

| **Study population and recruitment procedures**  Identification of Arms 1 and 2 (Tai Chi naïve older adults)  A total of 60 healthy older adults (50-79 years old) will be recruited for our study. Recruitment will target community dwellers living within the Greater Boston area. Identification of potential study participants will include a variety of procedures that we have successfully employed in our prior studies. These include: 1) Posting study brochures and flyers in ambulatory clinics associated with BIDMC’s Department of General Medicine and Primary Care and Department of Gerontology (approximately 4,000 and 1,000 patients, respectively); 2) Direct referrals from providers in these and other clinics with whom we have a long-standing working relationship (e.g. Dr. Lipsitz is the Chair of Gerontology and Drs. Yeh and Ahn are staff physicians in General Medicine); 3) Advertising in various publications including the Harvard Cooperative Program on Aging newsletter on healthy aging (disseminated 3 times a year to more than 9,500 older adults) and other local periodicals targeting older adults (e.g. *Fifty Plus Advocate, The Senior Times*), as well as on internet based resources such as Craig’s list; 4) BIDMC Syncope and Falls in the Elderly (SAFE) laboratory IRB approved database, whereby Dr. Novak provides names of potentially eligible participants who have offered to participate in SAFE lab studies; 5) Flyer placement in the communities surrounding the study’s network of Tai Chi schools (e.g., community bulletin boards, libraries).  Identification of Arm 3 (Tai Chi Expert Group)  A total of 30 healthy older adults (50-79 years old) each with over five years of tai chi practice will be recruited for our study. Recruitment will target tai chi experts living within the Greater Boston area. Potential study participants will be identified through advertisement and presentations given by PI and/or other co-investigator at Tai Chi schools.  Identification of Arm 4 (Young Comparison Group)  A total of 15 healthy young adults (25-35 years old) with no prior tai chi experience will be recruited for our study. Recruitment will target young adults living within the Greater Boston area. Potential study participants will be identified through advertisement and presentations given by PI and/or other co-investigator in the community.  5.2.2. Eligibility criteria  Arms 1 and 2: Recruitment will focus on healthy, relatively sedentary, older Tai Chi-naïve adults. Eligibility criteria will be based on screens conducted by study staff and confirmed by BIDMC GCRC nursing staff.  Arm 3 (Tai Chi Expert Group): Recruitment will focus on healthy tai chi experts with over five years of tai chi practice. Eligibility criteria will be based on screens conducted by study staff and confirmed by BIDMC GCRC nursing staff.  Arm 4 (Young Comparison Group): Recruitment will focus on healthy, relatively sedentary, young Tai Chi-naïve adults. Eligibility criteria will be based on screens conducted by study staff and confirmed by BIDMC GCRC nursing staff.  Arms 1 and 2 Inclusion criteria:  • Ages 50-79  • Living within the Greater Boston area  • Willing to adhere to 6 month Tai Chi training protocol  • Patients with a history of both solid and hematologic cancer (≥ 5 years of complete remission and who do not require chronic maintenance chemotherapy). Chronic use of hormonal agents such as aromatase inhibitors and GnRH analogs, however, are permissible with the understanding that these agents do not substantially alter the cardiovascular, autonomic, and postural control systems.  • Patients with prostate cancer (Stage I or II only)  • Patients with dermatological cancer that has been completely resected with no additional incidence.  Arm 3 (Tai Chi Expert Group) Inclusion criteria:  • Ages 50-79  • Have over five years of Tai Chi practice  • Patients with history of both solid and hematologic cancer (≥5 years of complete remission and who do not require chronic maintenance chemotherapy). Chronic use of hormonal agents such as aromatase inhibitors and GnRH analogs, however, are permissible with the understanding that these agents do not substantially alter the cardiovascular, autonomic, and postural control systems.  • Patients with prostate cancer (Stage I-II)  • Patients with dermatological cancer that has been completely resected with no additional incidence.  Arm 4 (Young Comparison Group) Inclusion criteria:  • Ages 25-35  • Living within the Greater Boston area  • Patients with a history of both solid and hematologic cancer (≥ 5 years of complete remission and who do not require chronic maintenance chemotherapy). Chronic use of hormonal agents such as aromatase inhibitors and GnRH analogs, however, are permissible with the understanding that these agents do not substantially alter the cardiovascular, autonomic, and postural control systems.  • Patients with prostate cancer (Stage I or II only)  • Patients with dermatological cancer that has been completely resected with no additional incidence.  Exclusion Criteria:  • Chronic medical conditions, including: cardiovascular disease (myocardial infarction, angina, atrial fibrillation, or presence of a pacemaker), stroke, respiratory disease requiring daily use of an inhaler, diabetes mellitus, malignancies (patients with diagnosis of active cancer < 5 years ago requiring ongoing chemotherapy or use of cytotoxic agents), neurological conditions (e.g., seizure disorder, Parkinson’s, peripheral neuropathy), or other neuromuscular or musculoskeletal (requiring chronic use of pain medication) disease  • Acute medical condition requiring hospitalization within the past 6 months  • Self-reported (current) smoking or alcohol/drug abuse  • Uncontrolled Hypertension (resting SBP > 160 or DBP > 100mm Hg)  • Abnormal heart rate (resting HR > 100 bpm; <50bpm)  • Abnormal ECG (supraventricular tachyarrhythmia, atrial fibrillation, significant ST wave abnormality, 2^nd^ and 3^rd^ degree heart block)  • Pregnancy  • Current use of prescription medications including cardio- or vaso-active drugs and medications that can affect autonomic function including Beta agonists and antagonists, drugs with anticholineric properties (e.g. tricyclic antidepressants or anti psychotics), and cholinesterase inhibitor^[[1]](#footnote-1)^  • Self-reported inability to walk continuously for 15 minutes unassisted^[[2]](#footnote-2)^  • Regular Tai Chi practice within past 5 years‡‡  • Regular participation in physical exercise on average 4 or more times per week‡‡  Arms 1 and 2 Recruitment, enrollment and randomization procedures  Potential participants who learn about our prospective study from brochures, referrals, or advertisements will be directed to call the study coordinator, who will conduct an initial eligibility screen by telephone, and schedule potentially eligible individuals for a visit at the BIDMC Clinic Research Center (CRC) for further screening. The coordinator will also discuss the randomization process and emphasize the 6 month commitment for those randomized to Tai Chi. Phone screen eligible individuals will be asked to visit at least one Tai Chi school prior to the on-site CRC screening visit. At the end of our initial phone screen, all participants will be informed about the Syncope and Falls in the Elderly (SAFE) Laboratory Research Recruitment Repository Database, and if they agree, their contact information will be given to Dr. Vera Novak. Dr. Vera Novak is the director of the SAFE Lab, PI of the Research Recruitment Repository Database, and a co-investigator on this study. Prior to baseline testing, individuals will undergo formal consenting following BIDMC and NIH guidelines. Following baseline testing, participants will be randomly assigned to one of two interventions, Tai Chi or wait list control.  Our study statistician, Dr. Roger Davis will be responsible for randomization procedures. We will stratify participants by age (50-59, 60-69, 70-79), generate treatment assignments using a permuted-blocks randomization scheme with randomly-varying block sizes, and seal the treatment assignments in opaque, sequentially numbered envelopes for each stratum. The use of stratification will limit the potential for confounding by age. Upon randomization, study staff will enter the participant’s name and medical record number into a randomization log. All randomization materials will be stored in a locked file, with access limited to non-blinded study personnel.  Arm 3 (Tai Chi Expert Group) Recruitment and enrollment procedures  Potential participants who learn about our prospective study from referrals or advertisements will be directed to call the study coordinator, who will conduct an initial eligibility screen by telephone, and schedule potentially eligible individuals for a visit at the BIDMC Clinic Research Center (CRC) for further screening.  Arm 4 (Young Comparison Group) Recruitment and enrollment procedures  Potential participants who learn about our prospective study will be directed to call the study coordinator, who will conduct an initial eligibility screen by telephone, and schedule potentially eligible individuals for a visit at the BIDMC Clinic Research Center (CRC) for further screening.  Remuneration  Arms 1 and 2: All parking expenses associated with CRC visits will be paid for by the study. Eligible and consented participants who complete outcome assessments will be paid $100 at study midpoint (3 months), and $100 at study endpoint (6 months) with a maximum compensation of $200. Participants who complete the screening process but are not eligible to participate further will receive $50.Tuition for all Tai Chi training over the 6-month study period will be paid for by the study. Participants in the control group will also be offered 3 months of free Tai Chi training at the completion of the study at their choice of school within our network.    Arm 3 (Tai Chi Expert Group) Remuneration:  All parking expenses associated with CRC visits will be paid for by the study. Eligible and consented participants who complete outcome assessments will be paid $200 following single visit.  Arm 4 (Young Comparison Group) Remuneration:  All parking expenses associated with CRC visits will be paid for by the study. Eligible and consented participants who complete outcome assessments will be paid $50 following single visit. |
| --- |

####

#### B4. POSSIBLE BENEFITS

| Because we will offer Tai Chi classes at the end of the study period to patients randomized to the waitlist control group, all patients who enroll in the study will receive Tai Chi instruction for free regardless of group. Preliminary research indicates that Tai Chi may improve age-related physiological decline. Some patients who receive Tai Chi in this clinical study may experience improved functional capacity and quality of life. Participants will also be remunerated for their time. More generally, information gained from this study will provide valuable knowledge about the physiologic processes related to aging, and how Tai Chi impacts these processes. |
| --- |

#### B5. POSSIBLE RISKS AND ANALYSIS OF RISK/BENEFIT RATIO

| Tai Chi is likely to be a relatively safe activity. There have been no reports of serious adverse effects in the literature. We experienced no adverse events in our pilot studies with heart failure and vestibulopathy populations. Additionally, no adverse events related to Tai Chi training have been reported in our current pragmatic trial with osteopenic women, which takes place within the network of community-based Tai Chi schools we will use in this proposed study. All schools in this network have plans in place to respond to medical emergencies.  Nevertheless, some potential expected risks to the subject are as follows:  a. The physical activity of Tai Chi training may cause muscle soreness in deconditioned individuals. In addition, shortness of breath, dizziness, or falls are possible. These risks are considered to be minimal and are addressed in the consent form.  b. During our functional testing, including prolonged gait dynamics and jumping, the possibility of fatigue or muscle strain in deconditioned individuals is possible. Cardiovascular and balance testing trials involving paced-breathing may cause temporary discomfort and/or lightheadedness. Participants will be instructed to rest whenever needed during all exercise capacity and strength tests, and spotters will be used for clinical balance tests. Falls are also possible during gait, balance and jumping tests. Patients undergoing exercise testing will be fully monitored and supervised at all times. If during testing, blood pressure exceeds 180/110, or there are concerning hypotensive episodes, testing will be stopped and our study MD, Dr. Yeh, will be contacted immediately to determine if further action is needed or if the participant’s physician needs to be contacted. Since testing is being done in a hospital outpatient setting, immediate access to emergency facilities and care is available if deemed necessary by the study physician. A summary of the incident will also be FAXED to the participant’s primary care physician.  c. Behavioral data collection involves virtually no risk, however, psychosocial tests may cause minor emotional distress. Because participants are free to stop at any time and will be reminded of this throughout the assessment process, the risk of distress is low. In our experience, both physical and behavioral tests are well tolerated and complications are extremely rare, especially in the relatively healthy population we are targeting in this study. These risks are addressed in the consent form.  d. Keeping all data in locked files or computers with carefully guarded access will minimize the risk of inadvertent release of participant data. Participant data will be stored in de-identified form and will be identified only by ID number. Corresponding participant names will be kept by the PI in a separate locked file.  Serious Adverse Events (SAEs)  SAEs that are unanticipated, serious, and possibly related to the study intervention will be reported to the IRB and NIH/NCCAM in accordance with requirements. Anticipated SAEs or those unrelated to the study intervention will be reported to the same individuals/entities in accordance with requirements.  **Overall, the potential risks to participants are modest and are likely to be viewed as reasonable in relation to the anticipated benefits to subjects and others.** |
| --- |

#### B6. RECRUITMENT AND CONSENT PROCEDURES

| **Recruitment**  5.2.1. Identification of Arms 1 and 2 (Tai Chi naïve older adults)  A total of 60 healthy older adults (50-79 years old) will be recruited for our study. Recruitment will target community dwellers living within the Greater Boston area. Identification of potential study participants will include a variety of procedures that we have successfully employed in our prior studies. These include: 1) Posting study brochures and flyers in ambulatory clinics associated with BIDMC’s Department of General Medicine and Primary Care and Department of Gerontology (approximately 4,000 and 1,000 patients, respectively); 2) Direct referrals from providers in these and other clinics with whom we have a long-standing working relationship (e.g. Dr. Lipsitz is the Chair of Gerontology and Drs. Yeh and Ahn are staff physicians in General Medicine); 3) Advertising in various publications including the HCPOA newsletter on healthy aging (disseminated 3 times a year to more than 9,500 older adults) and other local periodicals targeting older adults (e.g. *Fifty Plus Advocate, The Senior Times*) and internet resources such as Craig’s list.  Potential participants who learn about our prospective study from brochures, referrals, or advertisements will be directed to call the study coordinator, who will conduct an initial eligibility screen by telephone, and schedule potentially eligible individuals for a visit at the BIDMC Clinic Research Center (CRC) for further screening.  5.2.1. Identification of Arm 3 (Tai Chi Experts Group)  A total of 30 healthy older adults (50-79 years old) with over five years of tai chi practice will be recruited for our study. Potential study participants will be identified through advertisement and presentations given by PI and/or other co-investigator at Tai Chi schools.  Potential participants who learn about our prospective study from referrals or advertisements will be directed to call the study coordinator, who will conduct an initial eligibility screen by telephone, and schedule potentially eligible individuals for a visit at the BIDMC Clinic Research Center (CRC) for further screening.  5.2.1. Identification of Arm 4 (Young Comparison Group)  A total of 15 healthy young adults (25-35 years old) with no prior tai chi experience will be recruited for our study. Potential study participants will be identified through advertisement and presentations given by PI and/or other co-investigator in the community.  Potential participants who learn about our prospective study will be directed to call the study coordinator, who will conduct an initial eligibility screen by telephone, and schedule potentially eligible individuals for a visit at the BIDMC Clinic Research Center (CRC) for further screening.  **Consent**  Written informed consent will be obtained from each subject at entry into the study; i.e. during their first visit to the BIDMC CRC. Informed consent is obtained by the following process:  • The subject will be asked to review the study consent form;  • The PI or Co-Investigator (Co-I) will meet with the subject to review the form, to confirm the subject’s understanding of the study, and to answer any questions that the subject might have; and  • Once the subject demonstrates understanding of the study and agrees to participate in the study, the consent will be signed in the presence of the PI (or Co-I) and a witness.  **Subject Protection**  Subjects can withdraw from the trial at any time and they will be informed that the study is entirely voluntary. |
| --- |

## B7. STUDY LOCATION

| **Privacy**  Before starting the initial phone screen with the study coordinator, participants responding to advertising and expressing interest in the study will be asked if it is a good time to complete the screening. They will be told that they will be asked to provide personal information.  In person screening will be conducted in a patient room at the GCRC. All testing will be conducted in the SAFE (Syncope and Falls in the Elderly) Laboratory at the Harvard-Thorndike General Clinical Research Center, Gryzmish 818b. |
| --- |

## B8. DATA SECURITY

| All written data will be stored in a locked file cabinet in the PI’s office, which itself is locked whenever the PI, co-investigator, or research assistant are not in the office. The paper questionnaires used for this trial will have only coded information without any identifiers and will be also transferred to the password-protected database.  In addition, all computer files will be password protected. Our study database will not include data items that uniquely identify participants such as subject name, address, phone number, social security number and medical record number, but will be indexed by a study ID number. All data processed will be in the aggregate form and every individual will be given a unique ID number. The Master List will be on file in a locked cabinet in the Principal Investigator’s office. Electronic communication with outside collaborators involves only unidentifiable information. |
| --- |

## B9 Multi-Site Studies

| Is the BIDMC the coordinating site or is the BIDMC PI the lead investigator of the multi-site study?  Yes  No |
| --- |

**Literature Cited**

1. Brown AK, Liu-Ambrose T, Tate R, Lord SR. The effect of group-based exercise on cognitive performance and mood in seniors residing in intermediate care and self-care retirement facilities: a randomised controlled trial. *Br J Sports Med.* 2009;43:608-614.

2. Angevaren M, Aufdemkampe G, Verhaar HJ, Aleman A, Vanhees L. Physical activity and enhanced fitness to improve cognitive function in older people without known cognitive impairment. *Cochrane Database Syst Rev.* 2008:CD005381.

3. Lipsitz LA, Goldberger AL. Loss of 'complexity' and aging. Potential applications of fractals and chaos theory to senescence. *Jama.* 1992;267:1806-1809.

4. Lipsitz LA. Aging as a Process of Complexity Loss. In: Deisboeck TS KJ, ed. *Complex Systems Science in Biomedicine*: Springer 2006:641-654.

5. Hausdorff JM. Gait dynamics, fractals and falls: finding meaning in the stride-to-stride fluctuations of human walking. *Hum Mov Sci.* 2007;26:555-589.

6. Costa M, Goldberger AL, Peng CK. Multiscale entropy analysis of complex physiologic time series. *Phys Rev Lett.* 2002;89:068102.

7. Goldberger AL. Non-linear dynamics for clinicians: chaos theory, fractals, and complexity at the bedside. *Lancet.* 1996;347:1312-1314.

8. Goldberger AL. Giles f. Filley lecture. Complex systems. *Proc Am Thorac Soc.* 2006;3:467-471.

9. Huikuri HV, Makikallio TH, Airaksinen KE, et al. Power-law relationship of heart rate variability as a predictor of mortality in the elderly. *Circulation.* 1998;97:2031-2036.

10. Bigger JT, Jr., Steinman RC, Rolnitzky LM, Fleiss JL, Albrecht P, Cohen RJ. Power law behavior of RR-interval variability in healthy middle-aged persons, patients with recent acute myocardial infarction, and patients with heart transplants. *Circulation.* 1996;93:2142-2151.

11. Hausdorff JM, Rios DA, Edelberg HK. Gait variability and fall risk in community-living older adults: a 1-year prospective study. *Arch Phys Med Rehabil.* 2001;82:1050-1056.

12. Pikkujamsa SM, Makikallio TH, Sourander LB, et al. Cardiac interbeat interval dynamics from childhood to senescence : comparison of conventional and new measures based on fractals and chaos theory. *Circulation.* 1999;100:393-399.

13. Beckers F, Verheyden B, Aubert AE. Aging and nonlinear heart rate control in a healthy population. *Am J Physiol Heart Circ Physiol.* 2006;290:H2560-2570.

14. Peng CK, Mietus JE, Liu Y, et al. Quantifying fractal dynamics of human respiration: age and gender effects. *Ann Biomed Eng.* 2002;30:1-10.

15. Costa M, Priplata A, Lipsitz LA, et al. Noise and poise: enhancement of postural complexity in the elderly with a stochastic resonance- based therapy. *Eur Phys Letters.* 2007;77: 68008.68001-68008.68005.

16. Hausdorff JM, Mitchell SL, Firtion R, et al. Altered fractal dynamics of gait: reduced stride-interval correlations with aging and Huntington's disease. *J Appl Physiol.* 1997;82:262-269.

17. Lipsitz LA. Physiological complexity, aging, and the path to frailty. *Sci Aging Knowledge Environ.* 2004;2004:pe16.

18. Goldberger AL, Amaral LA, Hausdorff JM, Ivanov P, Peng CK, Stanley HE. Fractal dynamics in physiology: alterations with disease and aging. *Proc Natl Acad Sci U S A.* 2002;99 Suppl 1:2466-2472.

19. Taylor-Piliae RE. Tai Chi as an adjunct to cardiac rehabilitation exercise training. *J Cardiopulm Rehabil.* 2003;23:90-96.

20. Yeh G, Wood M, Lorell B, et al. Effects of Tai-Chi mind-body movement therapy on functional status and exercise capacity in patients with chronic heart failure: a randomized controlled trial. *Am J Med.* 2004;117:541-548.

21. Wu G. Evaluation of the effectiveness of Tai Chi for improving balance and preventing falls in the older population--a review. *J Am Geriatr Soc.* 2002;50:746-754.

22. Wayne P, Krebs D, Wolf S, et al. Can Tai Chi improve vestibulopathic postural control? *Arch Phys Med Rehabil.* 2004;85:142-152.

23. Wu G, Hitt J. Ground contact characteristics of Tai Chi gait. *Gait Posture.* 2005;22:32-39.

24. Li F, McAuley E, Peter D, Terry E, Chaumeton NR. Tai Chi Enhances Self-Efficacy and Exercise Behavior in Older Adults. *Journal of Aging and Physical Acitivity.* 2001;9:161-171.

25. Dechamps A, Bourdel-Marchasson I. Effects of Tai Chi exercises on self-efficacy and psychological health. *Edr Rev Aging Phys Act.* 2007;4:25-32.

26. Peng CK, Buldyrev SV, Hausdorff JM, et al. Non-equilibrium dynamics as an indispensable characteristic of a healthy biological system. *Integr Physiol Behav Sci.* 1994;29:283-293.

27. Report C.

28. Ahn AC, Tewari M, Poon CS, Phillips RS. The clinical applications of a systems approach. *PLoS Med.* 2006;3:e209.

29. Ahn AC, Tewari M, Poon CS, Phillips RS. The limits of reductionism in medicine: could systems biology offer an alternative? *PLoS Med.* 2006;3:e208.

30. Goldberger AL, Peng CK, Lipsitz LA. What is physiologic complexity and how does it change with aging and disease? *Neurobiol Aging.* 2002;23:23-26.

31. Rowe JW, Andres R, Tobin JD, Norris AH, Shock NW. The effect of age on creatinine clearance in men: a cross-sectional and longitudinal study. *J Gerontol.* 1976;31:155-163.

32. Kerstjens HA, Rijcken B, Schouten JP, Postma DS. Decline of FEV1 by age and smoking status: facts, figures, and fallacies. *Thorax.* 1997;52:820-827.

33. Verdu E, Ceballos D, Vilches JJ, Navarro X. Influence of aging on peripheral nerve function and regeneration. *J Peripher Nerv Syst.* 2000;5:191-208.

34. Davidson MB. The effect of aging on carbohydrate metabolism: a review of the English literature and a practical approach to the diagnosis of diabetes mellitus in the elderly. *Metabolism.* 1979;28:688-705.

35. Muller DC, Elahi D, Tobin JD, Andres R. The effect of age on insulin resistance and secretion: a review. *Semin Nephrol.* 1996;16:289-298.

36. Ryan SM, Goldberger AL, Ruthazer R, Mietus J, Lipsitz LA. Spectral analysis of heart rate dynamics in elderly persons with postprandial hypotension. *Am J Cardiol.* 1992;69:201-205.

37. Makikallio TH, Hoiber S, Kober L, et al. Fractal analysis of heart rate dynamics as a predictor of mortality in patients with depressed left ventricular function after acute myocardial infarction. TRACE Investigators. TRAndolapril Cardiac Evaluation. *Am J Cardiol.* 1999;83:836-839.

38. Arzeno NM, Kearney MT, Eckberg DL, Nolan J, Poon CS. Heart rate chaos as a mortality predictor in mild to moderate heart failure. *Conf Proc IEEE Eng Med Biol Soc.* 2007;2007:5051-5054.

39. Ho KK, Moody GB, Peng CK, et al. Predicting survival in heart failure case and control subjects by use of fully automated methods for deriving nonlinear and conventional indices of heart rate dynamics. *Circulation.* 1997;96:842-848.

40. Makikallio TH, Seppanen T, Airaksinen KE, et al. Dynamic analysis of heart rate may predict subsequent ventricular tachycardia after myocardial infarction. *Am J Cardiol.* 1997;80:779-783.

41. Makikallio TH, Ristimae T, Airaksinen KE, Peng CK, Goldberger AL, Huikuri HV. Heart rate dynamics in patients with stable angina pectoris and utility of fractal and complexity measures. *Am J Cardiol.* 1998;81:27-31.

42. Chaves PHM, Varadhan R, Lipsitz LA, et al. Physiologic complexity underlying heart rate dynamics and frailty status in community-dwelling older women. *J Amer Geriatr Soc.* 2008;56:1698-1703.

43. Cavanaugh JT, Guskiewicz KM, Giuliani C, Marshall S, Mercer V, Stergiou N. Detecting altered postural control after cerebral concussion in athletes with normal postural stability. *Br J Sports Med.* 2005;39:805-811.

44. Cavanaugh JT, Guskiewicz KM, Giuliani C, Marshall S, Mercer VS, Stergiou N. Recovery of postural control after cerebral concussion: new insights using approximate entropy. *J Athl Train.* 2006;41:305-313.

45. Hausdorff JM, Edelberg HK, Cudkowicz ME, Singh MA, Wei JY. The relationship between gait changes and falls. *J Am Geriatr Soc.* 1997;45:1406.

46. Hausdorff JM, Edelberg HK, Mitchell SL, Goldberger AL, Wei JY. Increased gait unsteadiness in community-dwelling elderly fallers. *Arch Phys Med Rehabil.* 1997;78:278-283.

47. Hausdorff JM, Lertratanakul A, Cudkowicz ME, Peterson AL, Kaliton D, Goldberger AL. Dynamic markers of altered gait rhythm in amyotrophic lateral sclerosis. *J Appl Physiol.* 2000;88:2045-2053.

48. Hausdorff JM, Schaafsma JD, Balash Y, Bartels AL, Gurevich T, Giladi N. Impaired regulation of stride variability in Parkinson's disease subjects with freezing of gait. *Exp Brain Res.* 2003;149:187-194.

49. Mandelbrot B.

50. Lipsitz LA. Dynamics of stability: the physiologic basis of functional health and frailty. *J Gerontol A Biol Sci Med Sci.* 2002;57:B115-125.

51. Peng C-K, Havlin S, Stanley HE, Goldberger AL. Quantification of scaling exponents and crossover phenomena in nonstationary heartbeat time series. *Chaos.* 1995;5:82-87.

52. Costa M, Goldberger AL, Peng CK. Multiscale entropy analysis of biological signals. *Phys Rev E Stat Nonlin Soft Matter Phys.* 2005;71:021906.

53. Norris PR, Anderson SM, Jenkins JM, Williams AE, Morris JA, Jr. Heart rate multiscale entropy at three hours predicts hospital mortality in 3,154 trauma patients. *Shock.* 2008;30:17-22.

54. Norris PR, Stein PK, Morris JA, Jr. Reduced heart rate multiscale entropy predicts death in critical illness: a study of physiologic complexity in 285 trauma patients. *J Crit Care.* 2008;23:399-405.

55. Arking.

56. Kaplan DT, Furman MI, Pincus SM, Ryan SM, Lipsitz LA, Goldberger AL. Aging and the complexity of cardiovascular dynamics. *Biophys J.* 1991;59:945-949.

57. Iyengar N, Peng CK, Morin R, Goldberger AL, Lipsitz LA. Age-related alterations in the fractal scaling of cardiac interbeat interval dynamics. *Am J Physiol.* 1996;271:R1078-1084.

58. Shumway-Cook.

59. Cavanaugh JT, Guskiewicz KM, Stergiou N. A nonlinear dynamic approach for evaluating postural control: new directions for the management of sport-related cerebral concussion. *Sports Med.* 2005;35:935-950.

60. Cavanaugh JT, Goldvasser D, McGibbon CA, Krebs DE. Comparison of head- and body-velocity trajectories during locomotion among healthy and vestibulopathic subjects. *J Rehabil Res Dev.* 2005;42:191-198.

61. Collins JJ, De Luca CJ. Open-loop and closed-loop control of posture: a random-walk analysis of center-of-pressure trajectories. *Exp Brain Res.* 1993;95:308-318.

62. Duarte M, Sternad D. Complexity of human postural control in young and older adults during prolonged standing. *Exp Brain Res.* 2008;191:265-276.

63. Hausdorff JM, Peng CK, Ladin Z, Wei JY, Goldberger AL. Is walking a random walk? Evidence for long-range correlations in stride interval of human gait. *J Appl Physiol.* 1995;78:349-358.

64. Hausdorff JM, Purdon PL, Peng CK, Ladin Z, Wei JY, Goldberger AL. Fractal dynamics of human gait: stability of long-range correlations in stride interval fluctuations. *J Appl Physiol.* 1996;80:1448-1457.

65. Jordan K, Challis JH, Newell KM. Long range correlations in the stride interval of running. *Gait Posture.* 2006;24:120-125.

66. Jordan K, Challis JH, Newell KM. Walking speed influences on gait cycle variability. *Gait Posture.* 2007;26:128-134.

67. Thurner S, Mittermaier C, Ehrenberger K. Change of complexity patterns in human posture during aging. *Audiol Neurootol.* 2002;7:240-248.

68. Collins JJ, De Luca CJ, Burrows A, Lipsitz LA. Age-related changes in open-loop and closed-loop postural control mechanisms. *Exp Brain Res.* 1995;104:480-492.

69. Barnett SR, Morin RJ, Kiely DK, et al. Effects of age and gender on autonomic control of blood pressure dynamics. *Hypertension.* 1999;33:1195-1200.

70. Lipsitz LA, Morin R, Gagnon M, Kiely D, Medina A. Vasomotor instability preceding tilt-induced syncope: does respiration play a role? *J Appl Physiol.* 1997;83:383-390.

71. Cavanaugh JT, Mercer VS, Stergiou N. Approximate entropy detects the effect of a secondary cognitive task on postural control in healthy young adults: a methodological report. *J Neuroeng Rehabil.* 2007;4:42.

72. Yogev-Seligmann G, Hausdorff JM, Giladi N. The role of executive function and attention in gait. *Mov Disord.* 2008;23:329-342; quiz 472.

73. Lacour M, Bernard-Demanze L, Dumitrescu M. Posture control, aging, and attention resources: models and posture-analysis methods. *Neurophysiol Clin.* 2008;38:411-421.

74. Tulppo MP, Hautala AJ, Makikallio TH, et al. Effects of aerobic training on heart rate dynamics in sedentary subjects. *J Appl Physiol.* 2003;95:364-372.

75. Heffernan KS, Sosnoff JJ, Jae SY, Gates GJ, Fernhall B. Acute resistance exercise reduces heart rate complexity and increases QTc interval. *Int J Sports Med.* 2008;29:289-293.

76. Priplata AA, Patritti BL, Niemi JB, et al. Noise-enhanced balance control in patients with diabetes and patients with stroke. *Ann Neurol.* 2006;59:4-12.

77. Wayne P, Kaptchuk T. Challenges inherent to Tai Chi research: Part I--Tai Chi as a complex multi-component intervention. *J Altern Complement Med.* 2008;14:95-102.

78. Wojtek C-Zao. *National Expert Meeting on Qi Gong and Tai Chi Consensus Report.* Urbana, IL: University of Illnois at Urbana-Champaign; Nov, 2005 2005.

79. Barnes PM, Powell-Griner E, McFann K, Nahin RL. Complementary and alternative medicine use among adults: United States, 2002. *Adv Data.* 2004:1-19.

80. Lan C, Lai J, Chen S. Tai Chi Chuan: an ancient wisdom on exercise and health promotion. *Sports Med.* 2002;32:217-224.

81. Li J, Hong Y, Chan K. Tai chi: physiological characteristics and beneficial effects on health. *Br J Sports Med.* 2001;35:118-156.

82. Sandlund E, Norlander T. The effects of Tai Chi Chuan relaxation and exercise on stress responses and well-being: an overview of research. *Int. J. Stress Manage.* 2000;7:139-149.

83. Wolf S, O'Grady M, Xu T. Tai Chi Chuan. In: Wainapel S, Fast A, eds. *Alternative Medicine in Rehabilitation: A Guide for Practitioners*. New York: Demos Press; 2002:99-139.

84. Taylor-Pilae R, Froelicher E. The effectiveness of Tai Chi exercise in improving aerobic capacity, a meta-analysis. *Journal of Cardiovascular Nursing.* 2004;19:48-57.

85. Yeh GY, Wang CC, Wayne PM, Phillips RS. The Effect of Tai Chi Exercise on Blood Pressure: A Systematic Review. *Preventive Cardiology.* 2008;11:82-89.

86. Lee MS, Pittler MH, Taylor-Piliae RE, Ernst E. Tai chi for cardiovascular disease and its risk factors: a systematic review. *J Hypertens.* 2007;25:1974-1975.

87. Taylor-Piliae RE. The effectiveness of Tai Chi exercise in improving aerobic capacity: an updated meta-analysis. *Med Sport Sci.* 2008;52:40-53.

88. Thornton EW. Tai Chi exercise in improving cardiorespiratory capacity. *Med Sport Sci.* 2008;52:54-63.

89. Lai JS, Lan C, Wong MK, Tenh SH. Two-year trends in cardiorespiratory function among older Tai Chi Chuan practitioners and sedentary subjects. *J Am Geriatr Soc.* 1995;43:1222-1227.

90. Barrow DE, Bedford A, Ives G, O'Toole L, Channer KS. An evaluation of the effects of Tai Chi Chuan and Chi Kung training in patients with symptomatic heart failure: a randomised controlled pilot study. *Postgrad Med J.* 2007;83:717-721.

91. Lan C, Chen SY, Wong MK, Lai JS. Tai chi training for patients with coronary heart disease. *Med Sport Sci.* 2008;52:182-194.

92. Lan C, Chen SY. The effect of Tai Chi on cardiorespiratory function in patients with coronary artery bypass surgery. *Med Sci Sports.* 1999;31:634-638.

93. Channer K, Barrow D, Barrow R, Osborne M, Ives G. Changes in haemodynamic parameters following Tai Chi Chuan and aerobic exercise in patients recovering from acute myocardial infarction. *Postgrad Med J.* 1996;72:349-351.

94. Wolf SL, O'Grady M, Easley KA, Guo Y, Kressig RW, Kutner M. The influence of intense Tai Chi training on physical performance and hemodynamic outcomes in transitionally frail, older adults. *J Gerontol A Biol Sci Med Sci.* 2006;61:184-189.

95. Audette JF, Jin YS, Newcomer R, Stein L, Duncan G, Frontera WR. Tai Chi versus brisk walking in elderly women. *Age Ageing.* 2006;35:388-393.

96. Vaananen J, Xusheng S, Wang S, Laitinen T, Pekkarinen H, Lansimies E. Taichiquan acutely increases heart rate variability. *Clin Physiol Funct Imaging.* 2002;22:2-3.

97. Lu WA, Kuo CD. The effect of Tai Chi Chuan on the autonomic nervous modulation in older persons. *Med Sci Sports.* 2003:1972-1975.

98. Wang C, Collet JP, Lau J. The effect of Tai Chi on health outcomes in patients with chronic conditions: a systematic review. *Arch Intern Med.* 2004;164:493-501.

99. Komagata S, Newton R. The Effectiveness of Tai Chi on Improving Balance in Older Adults: An Evidence-based Review. *Journal of Geriatric Physical Therapy.* 2003;26:9-16.

100. Wayne PM, Krebs DE, Wolf SL, et al. Can Tai Chi improve vestibulopathic postural control? *Arch Phys Med Rehabil.* 2004;85:142-152.

101. Harmer PA, Li F. Tai Chi and falls prevention in older people. *Med Sport Sci.* 2008;52:124-134.

102. Barnett A, Smith B, Lord SR, al. E. Community-based group exercise improves balance and reduces falls in at-risk older people: a randomized controlled trial. *Age Ageing.* 2003;32:407-414.

103. Li F, Harmer P, Fisher KJ, McAuley E. Tai Chi: improving functional balance and predicting subsequent falls in older persons. *Med Sci Sports Exerc.* 2004;36:2046-2052.

104. Li F, Harmer P, Fisher KJ, et al. Tai Chi and fall reductions in older adults: a randomized controlled trial. *J Gerontol A Biol Sci Med Sci.* 2005;60:187-194.

105. Wolf S, Barnhart H, Ellison G, Coogler C. The effect of Tai Chi Quan and computerized balance training on postural stability in older subjects. Atlanta FICSIT Group. Frailty and Injuries: Cooperative Studies on Intervention Techniques. *Phys Ther.* 1997;77:371-381.

106. Wolf S, Barnhart H, Kutner N, McNeely E, Coogler C, Xu T. Reducing frailty and falls in older persons: an investigation of Tai Chi and computerized balance training. *J Am Geriatr Soc.* 1996;44:489-497.

107. Voukelatos A, Cumming RG, Lord SR, Rissel C. A randomized, controlled trial of tai chi for the prevention of falls: the Central Sydney tai chi trial. *J Am Geriatr Soc.* 2007;55:1185-1191.

108. Lin MR, Hwang HF, Wang YW, Chang SH, Wolf SL. Community-based tai chi and its effect on injurious falls, balance, gait, and fear of falling in older people. *Phys Ther.* 2006;86:1189-1201.

109. Wolf S, Sattin R, Kutner N, O'Grady M, Greenspan A, Gregor R. Intense Tai Chi exercise training and fall occurrences in older, transitionally frail adults: a randomized controlled trial. *J Am Geriatr Soc.* 2003;51:1693-1701.

110. Choi JH, Moon JS, Song R. Effects of Sun-style Tai Chi exercise on physical fitness and fall prevention in fall-prone older adults. *J Adv Nurs.* 2005;51:150-157.

111. Nowalk MP, Prendergast JM, Bayles CM, D'Amico FJ, Colvin GC. A randomized trial of exercise programs among older individuals living in two long-term care facilities: the FallsFREE program. *J Am Geriatr Soc.* 2001;49:859-865.

112. Faber MJ, Bosscher RJ, Chin APMJ, van Wieringen PC. Effects of exercise programs on falls and mobility in frail and pre-frail older adults: A multicenter randomized controlled trial. *Arch Phys Med Rehabil.* 2006;87:885-896.

113. Jacobson B, Chen H, Cashel C. The effect of Tai Chi Chuan training on balance, kinesthetic sense, and strength. *Percept Mot Skills.* 1997;84:27-33.

114. Shih J. Basic Beijing twenty-four forms of T'ai Chi exercise and average velocity of sway. *Percept Mot Skills.* 1997;84:287-290.

115. Hain T, Fuller L, Weil L, Kotsias J. Effects of T'ai Chi on balance. *Arch Otol Head Neck Surg.* 1999;125:1191-1195.

116. Forrest WR. Anticipatory postural adjustment and T'ai Chi Ch'uan. *Biomed Sci Instrum.* 1997;33:65-70.

117. Wong A, Lin Y, Chou S, Tang F, Wong P. Coordination exercise and postural stability in elderly people: effect of Tai Chi Chuan. *Arch Phys Med Rehabil.* 2001;82:608-612.

118. Tsang WW, Hui-Chan CW. Standing balance after vestibular stimulation in Tai Chi-practicing and nonpracticing healthy older adults. *Arch Phys Med Rehabil.* 2006;87:546-553.

119. Tsang WW, Wong VS, Fu SN, Hui-Chan CW. Tai Chi improves standing balance control under reduced or conflicting sensory conditions. *Arch Phys Med Rehabil.* 2004;85:129-137.

120. Wu G, Liu W, Hitt J, Millon D. Spatial, temporal and muscle action patterns of Tai Chi gait. *J Electromyogr Kinesiol.* 2004;14:343-354.

121. Mao DW, Li JX, Hong Y. Plantar pressure distribution during Tai Chi exercise. *Arch Phys Med Rehabil.* 2006;87:814-820.

122. Mao DW, Hong Y, Li JX. Characteristics of foot movement in Tai Chi exercise. *Phys Ther.* 2006;86:215-222.

123. Mao DW, Li JX, Hong Y. The duration and plantar pressure distribution during one-leg stance in Tai Chi exercise. *Clin Biomech (Bristol, Avon).* 2006;21:640-645.

124. Chan SP, Luk TC, Hong Y. Kinematic and electromyographic analysis of the push movement in tai chi. *Br J Sports Med.* 2003;37:339-344.

125. Hass CJ, Gregor RJ, Waddell DE, et al. The influence of Tai Chi training on the center of pressure trajectory during gait initiation in older adults. *Arch Phys Med Rehabil.* 2004;85:1593-1598.

126. Gatts SK, Woollacott MH. How Tai Chi improves balance: biomechanics of recovery to a walking slip in impaired seniors. *Gait Posture.* 2007;25:205-214.

127. Gatts SK, Woollacott MH. Neural mechanisms underlying balance improvement with short term Tai Chi training. *Aging Clin Exp Res.* 2006;18:7-19.

128. Xu DQ, Li JX, Hong Y. Effect of regular Tai Chi and jogging exercise on neuromuscular reaction in older people. *Age Ageing.* 2005;34:439-444.

129. Tsang WW, Hui-Chan CW. Effects of tai chi on joint proprioception and stability limits in elderly subjects. *Med Sci Sports Exerc.* 2003;35:1962-1971.

130. Tsang WW, Hui-Chan CW. Effects of exercise on joint sense and balance in elderly men: Tai Chi versus golf. *Med Sci Sports Exerc.* 2004;36:658-667.

131. Fong SM, Ng GY. The effects on sensorimotor performance and balance with Tai Chi training. *Arch Phys Med Rehabil.* 2006;87:82-87.

132. Kirsteins A, Dietz F, Hwang S. Evaluating the safety and potential use of a weight-bearing exercise, Tai-Chi Chuan, for rheumatoid arthritis patients. *Am J Phys Med Rehabil.* 1991;70:136-141.

133. Zhang JG, Ishikawa-Takata K, Yamazaki H, Morita T, Ohta T. The effects of Tai Chi Chuan on physiological function and fear of falling in the less robust elderly: an intervention study for preventing falls. *Arch Gerontol Geriatr.* 2006;42:107-116.

134. Wolf SL, Barnhart HX, Kutner NG, McNeely E, Coogler C, Xu T. Reducing frailty and falls in older persons: an investigation of Tai Chi and computerized balance training. Atlanta FICSIT Group. Frailty and Injuries: Cooperative Studies of Intervention Techniques. *J Am Geriatr Soc.* 1996;44:489-497.

135. Verhoef MJ, Lewith G, Ritenbaugh C, Boon H, Fleishman S, Leis A. Complementary and alternative medicine whole systems research: beyond identification of inadequacies of the RCT. *Complement Ther Med.* 2005;13:206-212.

136. Ritenbaugh C, Verhoef M, Fleishman S, Boon H, Leis A. Whole systems research: a discipline for studying complementary and alternative medicine. *Altern Ther Health Med.* 2003;9:32-36.

137. Yeh G, Mietus JE, Peng C-K, et al. Enhancement of Sleep Stability with Tai Chi Exercise in Chronic Heart Failure: Preliminary Findings Using an ECG-Based Spectrogram Method  *Sleep Medicine* 2008;9:527-536.

138. Yeh GY, Wayne PM, Phillips RS. T'ai Chi exercise in patients with chronic heart failure. *Med Sport Sci.* 2008;52:195-208.

139. Hausdorff JM, Ashkenazy Y, Peng CK, Ivanov PC, Stanley HE, Goldberger AL. When human walking becomes random walking: fractal analysis and modeling of gait rhythm fluctuations. *Physica A.* 2001;302:138-147.

140. Novak V, Hu K, Vyas M, Lipsitz LA. Cardiolocomotor Coupling in Young and Elderly People. *J. of Gerontol Med Sci.* 2007;62A:86-92.

141. Manor B, Hu K, P. Z, et al. Altered control of postural sway following cerebral infarction: a cross-sectional analysis. *Neurology.* 2009. In Press.

142. Kang HG, Costa MD, Priplata AA, et al. Frailty and the Degradation of Complex Balance Dynamics during a Dual Task Protocol. *J. Gerontology.* In Press.

143. Yeh GY, Mietus JE, Peng CK, et al. Enhancement of sleep stability with Tai Chi exercise in chronic heart failure: preliminary findings using an ECG-based spectrogram method. *Sleep Med.* 2008;9:527-536.

144. Mietus JE, Peng C-K, Henry I, Goldsmith RL, Goldberger AL. The pNNx-files: Reexamining a widely-used heart rate variability measure. *Heart.* 2002;88:378-380.

145. Thomas R, JE M, CK P, AL G. An Electrocardiogram-based technique to assess Cardiopulmonary Coupling During Sleep. *Sleep* 2005;28:1151-1161.

146. Wayne PM, Scarborough DM, Krebs DE, et al. Tai Chi for vestibulopathic balance dysfunction: a case study. *Altern Ther Health Med.* 2005;11:60-66.

147. McGibbon C, Krebs D, Parker S, Scarborough D, Wayne P, Wolf S. Tai Chi and vestibular rehabilitation improve vestibulopathic gait via different neuromuscular mechanisms: preliminary report. *BMC Neurology.* 2005;5:3.

148. McGibbon C, Krebs D, Wolf S, Wayne P, Scarborough D, Parker S. Tai Chi and vestibular rehabilitation effects on gaze and whole-body stability. *J Vestib Res.* 2004;14:467-478.

149. McGibbon CA, Krebs DE, Puniello MS. Mechanical energy analysis identifies compensatory strategies in disabled elders' gait. *J Biomech.* 2001;34:481-490.

150. Riley PO, Benda BJ, Gill-Body KM, Krebs DE. Phase plane analysis of stability in quiet standing. *J Rehabil Res Dev.* 1995;32:227-235.

151. Huang NE, Shen Z, Long SR. The empirical mode of decompostion and the Hilbert spectrum for nonlinear and non-stationary time series. *Proc R Soc Med.* 1998;454:903-995.

152. Wayne P, Kaptchuk T. Challenges inherent to Tai Chi Research: Part II--Defining the intervention adn optimal study design. *J Altern Complement Med.* 2008;In Review:191-197.

153. Novak V, Novak P, Opfer-Gehrking TL, O'Brien PC, Low PA. Clinical and laboratory indices that enhance the diagnosis of postural tachycardia syndrome (POTS). *Mayo Clinic Proc.* 1998;73:1141-1150.

154. Novak V, Novak P, de Champlain J, Le Blanc AR, Martin R, Nadeau R. Influence of respiration on heart rate and blood pressure fluctuations. *J Appl Physiol.* 1993;74:617-626.

155. Sorond FA, Serrador JM, Jones RN, Shaffer ML, Lipsitz LA. The sit-to-stand technique for the measurement of dynamic cerebral autoregulation. *Ultrasound Med Biol.* 2009;35:21-29.

156. Fried LP, Tangen CM, Walston JD, et al. Frailty in older adults: evidence for phenotype. *J Gerontol Med Sci.* 2001;56A:M146-M156.

157. Beauchet O, Annweiler C, Allali G, Berrut G, Herrmann FR, Dubost V. Recurrent falls and dual task-related decrease in walking speed: is there a relationship? *J Am Geriatr Soc.* 2008;56:1265-1269.

158. Shinkai S, Watanabe S, Kumagai S, et al. Walking speed as a good predictor for the onset of functional dependence in a Japanese rural community population. *Age Ageing.* 2000;29:441-446.

159. Vereeck L, Wuyts F, Truijen S, Van de Heyning P. Clinical assessment of balance: normative data, and gender and age effects. *Int J Audiol.* 2008;47:67-75.

160. Kuh D, Bassey EJ, Butterworth S, Hardy R, Wadsworth ME. Grip strength, postural control, and functional leg power in a representative cohort of British men and women: associations with physical activity, health status, and socioeconomic conditions. *J Gerontol A Biol Sci Med Sci.* 2005;60:224-231.

161. Bosco C, Luhtanen P, Komi PV. A simple method for measurement of mechanical power in jumping. *Eur J Appl Physiol Occup Physiol.* 1983;50:273-282.

162. Fujita Y, Nakamura Y, Hiraoka J, et al. Physical-strength tests and mortality among visitors to health-promotion centers in Japan. *J Clin Epidemiol.* 1995;48:1349-1359.

163. Perry MC, Carville SF, Smith IC, Rutherford OM, Newham DJ. Strength, power output and symmetry of leg muscles: effect of age and history of falling. *Eur J Appl Physiol.* 2007;100:553-561.

164. Bean JF, Kiely DK, LaRose S, Alian J, Frontera WR. Is stair climb power a clinically relevant measure of leg power impairments in at-risk older adults? *Arch Phys Med Rehabil.* 2007;88:604-609.

165. Norkin CC WJ. *Measurement of Joint Motion: A Guide to Goniometry, 3rd Edition.* Los Angeles, CA: F.A. Davis Co; 2003.

166. Jones CJ, Rikli RE, Max J, Noffal G. The reliability and validity of a chair sit-and-reach test as a measure of hamstring flexibility in older adults. *Res Q Exerc Sport.* 1998;69:338-343.

167. Baumgartner.

168. Jones NL, Makrides L, Hitchcock C, Chypchar T, McCartney N. Normal standards for an incremental progressive cycle ergometer test. *Am Rev Respir Dis.* 1985;131:700-708.

169. Heil DP, Freedson PS, Ahlquist LE, Price J, Rippe JM. Nonexercise regression models to estimate peak oxygen consumption. *Med Sci Sports Exerc.* 1995;27:599-606.

170. Jackson AS, Beard EF, Wier LT, Ross RM, Stuteville JE, Blair SN. Changes in aerobic power of men, ages 25-70 yr. *Med Sci Sports Exerc.* 1995;27:113-120.

171. Bohannon RW. Hand-grip dynamometry predicts future outcomes in aging adults. *J Geriatr Phys Ther.* 2008;31:3-10.

172. Ware JE, Jr., Gandek B. Overview of the SF-36 Health Survey and the International Quality of Life Assessment (IQOLA) Project. *J Clin Epidemiol.* 1998;51:903-912.

173. Ware JE, Kosinski M. Interpreting SF-36 summary health measures: a response. *Qual Life Res.* 2001;10:405-413; discussion 415-420.

174. Bowie CR, Harvey PD. Administration and interpretation of the Trail Making Test. *Nat Protoc.* 2006;1:2277-2281.

175. Arbuthnott K, Frank J. Trail making test, part B as a measure of executive control: validation using a set-switching paradigm. *J Clin Exp Neuropsychol.* 2000;22:518-528.

176. Frazen MD, Paul D, Iverson G. Cross-validation of the alternate forms of reliability of the Trail Making Test. *Clin. Neuropsyhcol.* 1996;10:125-129.

177. Strauss E, Sherman E, Spreen. *A compnedium of neuropsychological tests: Administration, norms, and commentary (3rd ed.)*. New York: Oxford University Press; 2006.

178. Ross TP, Calhoun E, Cox T, Wenner C, Kono W, Pleasant M. The reliability and validity of qualitative scores for the Controlled Oral Word Association Test. *Arch Clin Neuropsychol.* 2007;22:475-488.

179. Ross TP, Furr AE, Carter SE, Weinberg M. The psychometric equivalence of two alternate forms of the Controlled Oral Word Association Test. *Clin Neuropsychol.* 2006;20:414-431.

180. Folstein MF, Folstein SE, McHugh PR. "Mini-mental state". A practical method for grading the cognitive state of patients for the clinician. *J Psychiatr Res.* 1975;12:189-198.

181. Rovner BW, Folstein MF. Mini-mental state exam in clinical practice. *Hosp Pract (Off Ed).* 1987;22:99, 103, 106, 110.

182. Tangalos EG, Smith GE, Ivnik RJ, et al. The Mini-Mental State Examination in general medical practice: clinical utility and acceptance. *Mayo Clin Proc.* 1996;71:829-837.

183. Weissman MM, Sholomskas D, Pottenger M, Prusoff BA, Locke BZ. Assessing depressive symptoms in five psychiatric populations: a validation study. *Am J Epidemiol.* 1977;106:203-214.

184. Himmelfarb S, Murrell SA. Reliability and validity of five mental health scales in older persons. *J Gerontol.* 1983;38:333-339.

185. McNair DM, Lorr M, Droppelman LF. *Manual for the profile of mood states*. San Diego: EDITS Educational and Industrial Testing Services, Inc.; 1992.

186. Brown D, Wang Y, Ward A, et al. Chronic psychological effects of exercise and exercise plus cognitive strategies. *Med Sci Sports Exerc.* 1995;27:765-775.

187. Kalauokalani D, Cherkin DC, Sherman KJ, Koepsell TD, Deyo RA. Lessons from a trial of acupuncture and massage for low back pain: patient expectations and treatment effects. *Spine (Phila Pa 1976).* 2001;26:1418-1424.

188. Myers SS, Phillips RS, Davis RB, et al. Patient expectations as predictors of outcome in patients with acute low back pain. *J Gen Intern Med.* 2008;23:148-153.

189. Hong SL, Manor B, Li L. Stance and sensory feedback influence on postural dynamics. *Neurosci Lett.* 2007;423:104-108.

190. Costa M, Peng CK, Goldberger AL, Hausdorff JM. Multiscale entropy analysis of human gait dynamics. *Physica A.* 2003;330:53-60.

191. Costa MD, Peng CK, Goldberger AL. Multiscale Analysis of Heart Rate Dynamics: Entropy and Time Irreversibility Measures. *Cardiovasc Eng.* 2008.

192. Richman JS, Lake DE, Moorman JR. Sample entropy. *Methods Enzymol.* 2004;384:172-184.

193. Judd CA, Kenny DA. Process analysis: estimating mediation in evaluation research. *Evaluation Research.* 1981;5:602-619.

1. Use of beta blockers for hypertension will be allowed in Arm 3 (Tai Chi Expert Group) [↑](#footnote-ref-1)
2. Not applicable to Arm 3 (Tai Chi Expert Group) [↑](#footnote-ref-2)
